# Gauging the importance of structural parameters for hyperfine coupling constants in organic radicals†

**DOI:** 10.1039/d3ra02476h

**Published:** 2023-05-12

**Authors:** Conrad Szczuka, Rüdiger-A. Eichel, Josef Granwehr

**Affiliations:** a Institute of Energy and Climate Research (IEK-9), Forschungszentrum Jülich GmbH 52425 Jülich Germany co.szczuka@fz-juelich.de; b Institute of Physical Chemistry, RWTH Aachen University 52056 Aachen Germany; c Institute of Technical and Macromolecular Chemistry, RWTH Aachen University 52056 Aachen Germany

## Abstract

The identification of fundamental relationships between atomic configuration and electronic structure typically requires experimental empiricism or systematic theoretical studies. Here, we provide an alternative statistical approach to gauge the importance of structure parameters, *i.e.*, bond lengths, bond angles, and dihedral angles, for hyperfine coupling constants in organic radicals. Hyperfine coupling constants describe electron–nuclear interactions defined by the electronic structure and are experimentally measurable, for example, by electron paramagnetic resonance spectroscopy. Importance quantifiers are computed with the machine learning algorithm *neighborhood components analysis* using molecular dynamics trajectory snapshots. Atomic–electronic structure relationships are visualized in matrices correlating structure parameters with coupling constants of all magnetic nuclei. Qualitatively, the results reproduce common hyperfine coupling models. Tools to use the presented procedure for other radicals/paramagnetic species or other atomic structure-dependent parameters are provided.

## Abstract 2

Machine learning approach to identify dependencies between the structure of radicals and their underlying hyperfine coupling constants. Universally applicable also for other atomic parameters.

## Graphical abstract

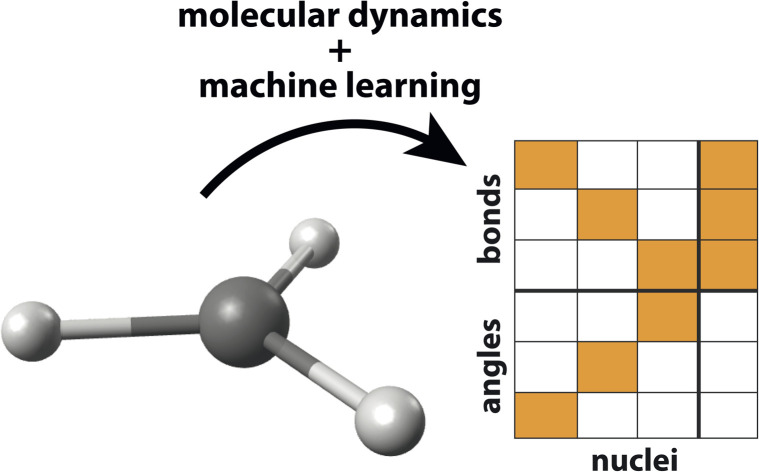

## 1 Introduction

Machine learning techniques are increasingly applied in chemical sciences.^1^ Applications can be roughly classified into predictions of properties based on atomic/electronic structure^2^ and (statistical) evaluation of experimental results.^1^ In the subfield of electron magnetic resonance, applications focus on the interpretation of acquired spectra, *e.g.*, from continuous-wave electron paramagnetic resonance (EPR),^3^ double electron–electron resonance (DEER),^4^ electron–nuclear double resonance (ENDOR),^5^ or hyperfine sublevel correlation spectroscopy (HYSCORE).^6^

Most machine learning techniques are prediction models that can predict a numerical output value by regression or classification based on input values (features). To improve model stability and accuracy, the dimensionality of feature sets can be reduced through preprocessing, *e.g.*, by transformation into a new basis or selecting features through ranking their importance in determining the output. Neighborhood component analysis (NCA) is one example for a non-paramagnetic feature selection algorithm introduced by Goldberger *et al.*^7^ It was improved through introduction of a regularization term by Yang *et al.*,^8^ and validated in real-world applications.^9,10^ This algorithm can be used for feature selection but also simply to provide a gauge for importance of a feature for a particular response, as will be exploited herein.

Frequently targeted spectroscopic parameters of radicals and paramagnetic centers are hyperfine coupling constants, resulting from electron–nuclear interactions, that can be used to evaluate the underlying atomic and electronic structure. Hyperfine couplings arise from a non-zero spin density distribution evoked by spin delocalization and polarization. In small systems, some origins of hyperfine interactions based on, *e.g.*, exchange interactions or hyperconjugation, were identified.^11–14^ These mechanisms are often described locally for simplicity; however, the entirety of the electronic structure dictated by the type and configuration of all atoms defines the spin density distribution and, thereby, hyperfine coupling magnitudes.

Hyperfine couplings are described by an anisotropic rank 2 tensor ***A*** with three principal components *A*_*x*,*y*,*z*_ and tr(***A***) = *A*_iso_.^15^ Since interpretation of experimental *A*_*x*,*y*,*z*_ values is non-trivial, a comparison with calculated values from *ab initio* models is usually performed.^16–19^ Sufficiently accurate calculations are often possible, but require incorporation of electron correlation defined by the used electronic theory, solvation effects defined by the structural model, and dynamic contributions. Dynamics can be included by averaging computed hyperfine coupling constants from molecular dynamics snapshots,^20,21^ since molecular motion on timescales of fs is fast compared to the experimental time scale of ps to ns for EPR. Trajectory analyses generally show a non-Gaussian coupling constant distribution in histograms.^21,22^ In principle, these trajectories and the computed coupling constants contain statistical information on structure–hyperfine relations that are usually removed through the averaging, but will be exploited herein.

In this work, molecular dynamics trajectories are used to derive correlations between molecular structure and hyperfine coupling constants of common organic radicals using machine learning. Correlations are quantified by feature weights calculated using the NCA algorithm. Features involve all bonds, angles, and dihedrals extracted from the trajectory's coordinates, and targeted responses are *A*_*x*,*y*,*z*,iso_. First, adequate simulation parameters are derived using the principle of magnetic equivalence. Secondly, example radicals are analyzed regarding structure–hyperfine relations extracted by the algorithm. The discussion is complemented by considering the structure, spin density distribution, hyperfine tensor orientations/magnitudes, and links to relations known in literature.

## 2 Methods

### 2.1 Density functional theory calculations

Density functional theory (DFT) calculations were conducted using *ORCA 5.0.1*.^23^ Organic radicals were geometry optimized using the hybrid functional B3LYP^24,25^ and def2-TZVP^26^ basis sets. XYZ files are accessible in the data repository found at https://doi.org/10.26165/JUELICH-DATA/UH0LM2. The *ORCA*-MD module^27^ was used to calculate *ab initio* molecular dynamics (MD) trajectories, with velocities initialized assuming a temperature of 900 K or, for temperature comparison, in a range of 150 to 2000 K. Initialized temperatures were maintained using a Nosé–Hoover chains^28–30^ (NHC) thermostat with a time constant of 20 fs. Time steps of 0.5 fs were applied to address high-frequency hydrogen motion. For MD snapshots in random steps of 0.5–40 fs along the trajectory, hyperfine coupling tensors for ^1^H, ^13^C, and ^17^O were calculated using B3LYP and EPR-III (ref. 31) basis sets, disregarding contributions from spin–orbit coupling. Hyperfine tensors are referenced to the *g*-tensor frame. Example ORCA input files are given in the ESI, Section F.†

### 2.2 Data processing workflow

Further data processing and analysis was performed in *MATLAB* R2021b using the *Statistics and Machine Learning Toolbox* and *Deep Learning Toolbox*, involving (a) conversion of *xyz* coordinates into position-independent structure parameters comprising bonds, angles, and dihedrals, and (b) NCA to identify structure parameter importance for changes in hyperfine coupling constants. The *MATLAB* scripts are available online (https://doi.org/10.26165/JUELICH-DATA/UH0LM2).

#### 2.2.1 Structure parameter extraction

MD *xyz* trajectory files as outputted by *ORCA* are used for parameter extraction. The first structure, which is the geometry optimized structure, is analyzed to yield atomic distances with an upper cutoff of 1.5 Å to only include chemical bonds. These bonds are assigned to atomic identifiers since MD snapshots might comprise other non-chemical-bond distances <1.5 Å along the trajectory. Angles are extracted based on bond connectivities. Dihedrals are specified by a chain of four bonded atoms where one dihedral per set of two central atoms is defined. To use structure parameters as machine learning features, dihedral angles are transformed to cosine and sine values to account for rotational symmetry, *i.e.*, circumvent problems with the parameter discontinuity at 360° = 0°. Bond angles are given in degrees to reduce the total number of features. Bonds are given in Å.

#### 2.2.2 Neighborhood components analysis

The NCA^7,8^ algorithm (*fscnca* in *MATLAB, Statistics and Machine Learning Toolbox*) is used to extract importance values of specific features for a resulting response. Features imply all extracted structure parameters as explained in Structure parameter extraction (2.2.1). Responses are *A*_*x*_, *A*_*y*_, *A*_*z*_, or *A*_iso_. As hyperfine interactions are performed for all magnetic nuclei, importance values form a 3D matrix per molecule. The regularization parameter *λ* was either optimized using the Limited memory Broyden–Fletcher–Goldfarb–Shanno^32^ (LBFGS) algorithm and fourfold cross-validation, or it was set to 0.05, as indicated. Features were individually standardized, *i.e.*, transformed to numbers ranging from 0 to 1, ensuring comparability albeit using a single *λ*.

## 3 Results and discussion

### 3.1 Workflow

**Fig. 1:**
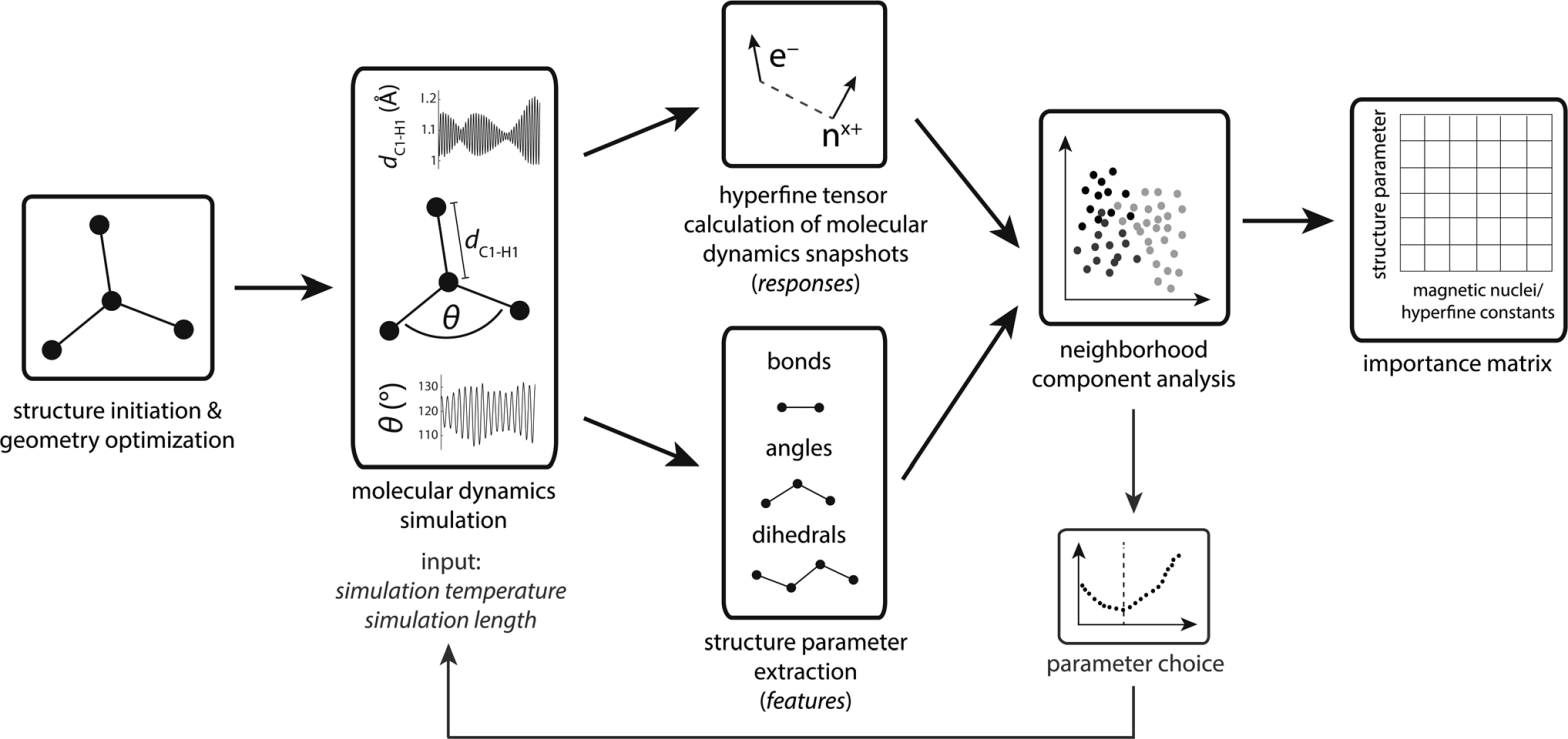
Workflow chart of the approach taken in this work.

The workflow is schematized in Fig. 1. First, organic radicals (Table 1) are initialized as xyz structures, pre-optimized using force-fields, and ultimately optimized with density functional theory. These structures serve as input for *ab initio* molecular dynamics (MD) simulations using a simulation temperature of 900 K, chosen based on results presented in the section *Parameter choice* below. Up to 30 ps MD simulation trajectories were computed to provide sufficient statistics for the machine learning algorithm. To account for the non-linear increase of structure parameters with the number of atoms in a molecule, the MD step number lies between 20 000 (≙10 ps) for the methyl and 60 000 (≙30 ps) for the tryptophan-type radical, compromising between necessary statistics and computation resources. MD trajectories represent the computationally most demanding step, using more than 93% of the computational resources (Table S1†). Along MD trajectories, snapshots were chosen for hyperfine coupling tensor calculations. We deliberately chose not to calculate hyperfine tensors for every step to reduce structural similarity between single data points. Because structure parameters oscillate predominantly according to a superposition of regular sine waves,^33^ choosing snapshots in regular intervals will significantly decrease specific structure parameter variations if the interval corresponds to the frequency of a normal mode. Thus, we use an evenly distributed randomized step interval of 1–80 for hyperfine coupling calculations.

**Table 1:** List of investigated organic radicals, calculation parameters, and structure parameters

Radical	# of MD steps	# of hyperfine calculations	# of bonds	# of angles	# of dihedrals
Methyl	20 000	505	3	3	0
Ethyl	50 000	1250	6	9	1
Methyl peroxy	40 000	969	5	7	1
Semiquinone	60 000	1496	13	19	7
Tyrosyl (see ESI)	70 000	1715	15	24	7
Tryptophan-type (see ESI)	60 000	1466	19	31	11

### 3.2 Neighborhood components analysis

The hyperfine coupling constants can be described as output (response variable) given a defined set of intrinsic variables including distances (chemical bonds), angles, and dihedrals. These structure parameters can serve as features for the machine learning algorithm NCA.^7,8^ NCA mathematically maps the input features to the outputs by generating weights for each feature. The feature weights determine the numerical importance of each feature in this mapping. Intuitively, features with higher weights contribute more to the output than features with lower weights. For stochastically fluctuating features, regularization is a strategy to prevent the model from overaccurately tracing these fluctuations, which could lead to unphysical results that would be referred to as overfitting. Regularization penalizes feature weights of excessive value by adding a term to the error estimation between the real outputs and the model prediction. In NCA,^8^ the penalty term consists of the squared weights multiplied with a regularization parameter *λ*. Herein, *λ* = 0.05 is chosen based on results from the section *Parameter choice* below.

To improve the accuracy of machine learning algorithms, choice and quality of features are crucial.^34^ Herein, features are selected as follows. In contrast to bond lengths being continuous variables, dihedrals are circular variables exhibiting a discontinuity, but can be converted to continuous sine and cosine values. Bond angles are not converted likewise since they are defined in the interval [0°, 180°] and thus, are pseudo-continuous. Another possibility to reduce the number of features is to consider overlapping dihedrals. Dihedrals are described by four chemically connected atomic positions. For a standard descriptive example like 1,2-dichloroethane, typically a single dihedral is used, assuming that the other atomic positions can be inferred by symmetry. However, for randomly moving atoms in molecular dynamics, all existing sets of four connected atoms need to be considered. Yet, we expect the additional dihedrals to be highly correlated. Hence, only one dihedral per two defining central atoms is kept as a feature. Before feeding the NCA algorithm, each feature is individually standardized to have zero mean and unit standard deviation, avoiding artefacts from different unit scales and variation magnitudes. This strategy for standardization is ideally suited for continuous and Gaussian-like distributed features, which are often observed in MD trajectories, particularly for bonds and angles.^21,22,35,36^ Other standardization strategies might be superior depending on the conformational energy landscape.

**Fig. 2:**
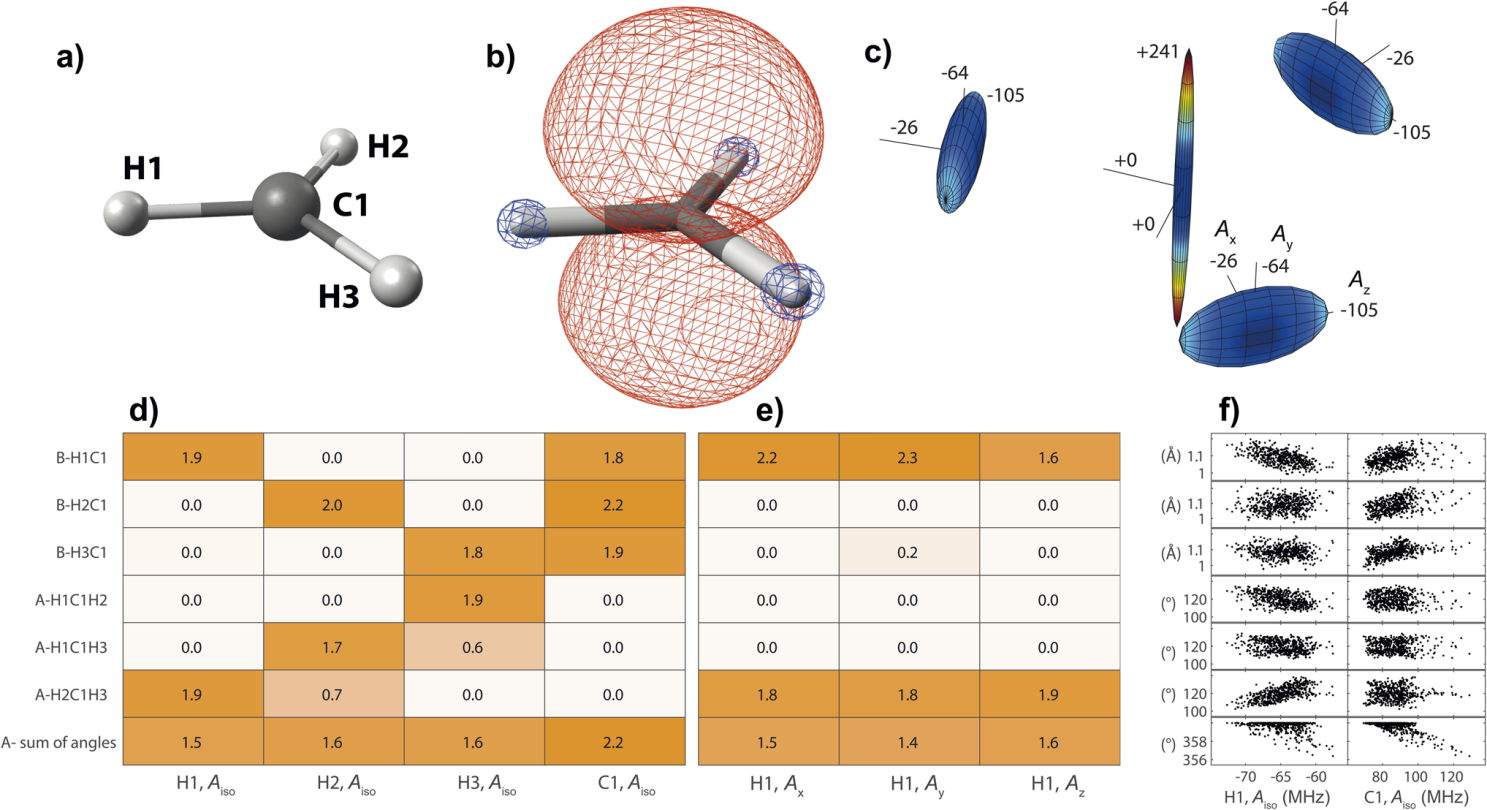
Structure–hyperfine relationship for the methyl radical with atomic labels given in (a). (b) For the geometry optimized structure, positive (red) and negative (blue) spin density is displayed at an iso-value of 0.007/*a*_0_^3^, where *a*_0_ is the Bohr radius. (c) Hyperfine coupling tensors are visualized for each magnetic nucleus, normalized to the largest overall coupling constant. Importance matrices from neighborhood components analysis using *λ* = 0.05 are given for *A*_iso_ of all nuclei (d), and for all hyperfine tensor principal components of H1 (e). Structure parameters are classified as bond lengths (B) and angles (A). (f) Raw data plots including all 505 hyperfine calculations.

On the basis of the created feature set, NCA can be performed for an arbitrary response variable that depends on the features. Herein, we include hyperfine coupling constants *A*_*x*,*y*,*z*,iso_ for all magnetic nuclei in a given organic radical. A simple example is the methyl radical with atomic labels, spin density, and hyperfine tensor plot depicted in Fig. 2a–c, respectively. The importance matrix regarding the central ^13^C and all three ^1^H nuclei shows clear non-zero entries (Fig. 2d and e). *A*_iso_ correlations are also evident from visible inspection of the raw data plotted in Fig. 2f, confirming NCA-derived correlations. For the response *A*_iso_(H1), positive correlation with the angle H2C1H3 opposite of H1 and negative correlations with the bond length H1C1 and the sum of all bond angles are inferred. For *A*_iso_(C1), positive correlation with all bond lengths and negative correlation with the sum of all angles is inferred. Without discussing chemical and physical origins at this point, a set of symmetry arguments are evident for the matrix in Fig. 2d. Due to magnetic equivalence of H1, H2, and H3, the upper left 3 × 3 matrix characterizing bonds and the adjacent lower 3 × 3 matrix characterizing angles should ideally be centrosymmetric. Similarly, all hydrogen atoms should be equally important for *A*_iso_(C1), so importance values for bonds and angles should be identical. Deviations from these symmetry considerations, like the non-centrosymmetric entries with importance values below one, are assumed to stem from insufficient statistics along the MD trajectory.

### 3.3 Parameter choice

The chosen MD parameters have a large influence on the dataset quality for NCA. Since the importance matrix elements are unknown *a priori*, symmetry arguments as introduced in the last section are exploited to gauge parameter suitability. More specifically, for H atom symmetry, the first three matrix columns from Fig. 2d are permuted to meet the symmetry arguments, *i.e*., the first entry of nucleus H*X*, with *X* ∈ {1, 2, 3}, corresponds to the bond H*X*C1 and the fourth entry corresponds to the opposite angle not involving H*X*. Permutations are performed for *A*_*x*,*y*,*z*_, and the cumulative mean square error comparing each pair of the {H1, H2, H3} group is calculated. For C atom symmetry, the standard deviation of the first three bond-associated entries and the following three angle-associated entries are calculated for *A*_*x*,*y*,*z*_, and the overall mean standard deviation is computed.

**Fig. 3:**
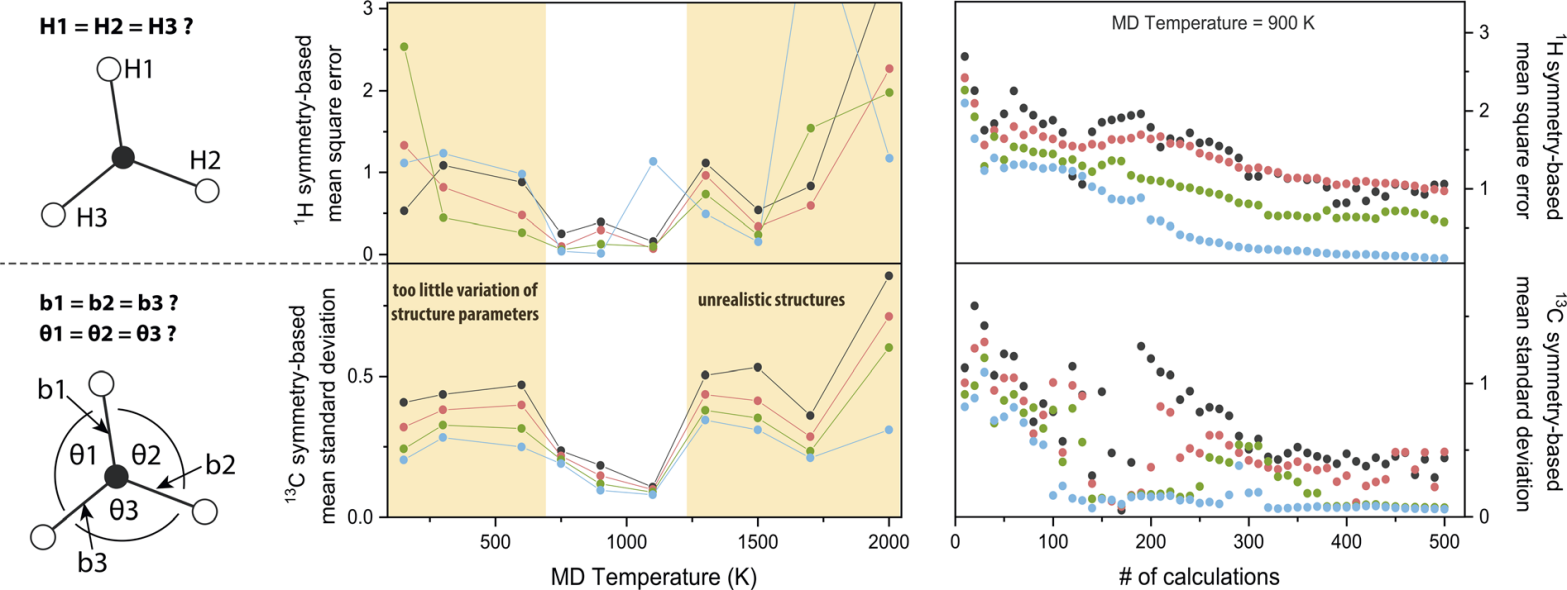
Symmetry-based analysis of molecular dynamics parameters determining the quality of methyl radical importance matrices. Symmetry arguments for ^1^H and ^13^C hyperfine couplings are visualized on the left. Design of symmetry-based descriptors is described in the section *Parameter choice* and plotted for *λ* = 0.03 (black), 0.05 (red), 0.08 (green), and 0.12 (blue). In the middle panel, the molecular dynamics temperature is optimized. In the right panel, error reduction with dataset size is analyzed.

Using these symmetry-based descriptors, the MD temperature *T*_MD_ is investigated (Fig. 3, central panel, see also Fig. S1†). For 0.03 ≤ *λ* ≤ 0.12, a window for minimum error is evident from roughly 700–1200 K, both for H and C symmetry gauges. By increasing *T*_MD_, the amplitude of structure parameter variations increases concomitantly. For example, the maximum amplitude of bond stretching/compression is 0.08 Å at 150 K and 0.26 Å at 2000 K. Maximum amplitude of angle stretching/compression is 7° at 150 K and 32° at 2000 K. Results suggest that in the low-to-intermediate *T*_MD_ regime, an increase in *T*_MD_ benefits the accuracy of the model, because the numerical spread of features/responses and sampling of the conformational space is enhanced. However, in the intermediate-to-high *T*_MD_ regime, the trend is reversed. Extreme structure parameters might lead to unforeseen high-temperature effects on the electronic structure, *e.g.* relatively strong hydrogen 1s orbital overlap for angles <90° observed for *T*_MD_ = 2000 K, resulting in alterations or discontinuities of the structure–hyperfine relationship. Deviations from realistic chemical structures using *T*_MD_ = 1500 K were also reported^37^ and above 2000 K pyrolysis of hydrocarbons occurs.^38^ We note that importance values may be slightly dependent on *T*_MD_, which is however most likely of minor significance for the semi-quantitative findings presented herein (Fig. S1†).

In Fig. 3 (right panel), the symmetry-based descriptors are used to investigate the dataset size *N*. For increasing *N*, statistics become better and correlations between atomic movements are averaged out. For high *λ* values, errors are generally smaller because weakly correlated features are forced to an importance value of zero. Equilibration of the descriptors for this trajectory is achieved with around *N* ≈ 350. Although minimization of the error is the ideal performance indicator, the importance matrix is already qualitatively reproduced with far smaller *N*; the underlying symmetry is indicated already for *N* = 25 (Fig. S1†) where the absolute importance values still deviate significantly from *N* = 505 (Fig. 2), but *correlated* or *uncorrelated* classification is already satisfying. Using rough extrapolation, dataset sizes were increased for increasing amounts of magnetic nuclei in a radical (Table 1). To compromise with available computational resources, the *N*-to-feature ratio decreases from 84 for the methyl radical to 24 for the tryptophan-type radical.

Lastly, the regularization parameter *λ* needs to be chosen appropriately, with feature weights approaching zero for *λ* → ∞, but a too small *λ* over-interprets the data (overfitting). *λ* can be tuned by optimizing the accuracy of the NCA model using cross-validation, where parts of the data are not used to train the model but to later validate its accuracy. Prediction accuracy optimization (Fig. S2†) for the methyl, ethyl, and methyl peroxy radicals was performed for each coupling constant individually, resulting in 0.01 ≤ *λ* ≤ 0.26 with an average of 0.07 and a median of 0.03 (Table S2†). The optimized *λ* values are very similar for magnetically equivalent nuclei and vary for different coupling magnitudes and nucleus type. Based on these test sets and to ensure better comparability in importance matrices, *λ* was chosen to be 0.05, in between the average and median for all figures in the main text. Additional figures with *λ* = 0.025 and 0.100 for all radicals are provided in the ESI, Section E.†

### 3.4 Chemical implications

In this section, organic radicals (Table 1) are investigated as test cases for the introduced feature design and NCA algorithm. In analogy to the methyl radical, importance matrices can be cross-checked by symmetry arguments. On that basis, the relationship of chemical information with atomic and electronic structure will be extracted, since hyperfine coupling constants directly result from the spin density distribution that is a function of the molecular orbital structure. The spin–orbit coupling contribution is disregarded in this work, as is common for organic radicals,^16^ saving computational resources and removing selection biases since spin–orbit coupling is significantly DFT-functional dependent.^39,40^ To reduce complexity, we will largely base the discussion on *A*_iso_ values.

#### 3.4.1 Methyl radical

The methyl radical 
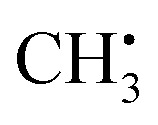
 is an experimentally^41^ and theoretically^42,43^ well-characterized organic radical with a carbon 2p_*z*_ orbital centered free electron (Fig. 2b), largely defining the anisotropy and magnitude of the ^13^C coupling tensor (Fig. 2c). In addition, spin density can spread over the molecule *via* spin polarization. For 
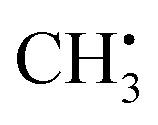
, exchange interactions between the (π)^1^ and (σ_CH_)^2^ orbital result in negative spin density around H atoms through different potentials felt by electrons with parallel and anti-parallel spin (*McConnell*^11^ exchange), indicated by a H Mulliken spin population of −0.03 for the relaxed structure. The anisotropy of the ^1^H hyperfine tensors is affected by through-space contributions; particularly from positive spin density around the central ^13^C,^44^ which explains less negative *A*_*x*,*y*_ that is pointing toward the C atom and parallel to the ^13^C hyperfine *z*-axis, respectively.

The importance matrix shows that *A*_iso_(H*X*) is correlated with the respective H*X*C1 bond length, likely increasing negative spin density at the nucleus through growing overlap between (π)^1^ and (σ_CH_)^2^, and thus increasing the exchange interaction. Analogously, *A*_iso_(C1) is affected by contributions from all bonds simultaneously. Regarding bond angles, *A*_iso_(H*X*) correlation with the opposite-lying angle is observed, *e.g.* H2C1H3 for *A*_iso_(H1), verified by symmetry arguments. Angles deviating from 120° likely alter the orbital configuration with respect to hybridization and inter-(σ_C1H*X*_)^2^ overlap, where larger H–H distances were found to result in more negative *A*_iso_.^45^ Consequently, also the two remaining angles should affect *A*_iso_. However, the opposite-lying angle is the only angle where both other H atoms either move further away or get closer concurrently to a reference ^1^H nucleus. In contrast, if H1C1H2 got larger, H1C1H3 could get larger as well, amplifying the effect on *A*_iso_(H1), or it could get smaller, compensating the effect. For 
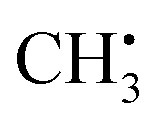
, the most analyzed structural deformation in literature is a trigonal pyramidal distortion, characterized by the angle of the H atom's positions and the relaxed structure's plane.^43,46^ To gauge this distortion, the *sum of all angles* was added as a feature herein and shows likewise correlation with all *A*_iso_(H*X*), evoked by moving ^1^H away from the nodal plane toward the 2p_*z*_ orbital lobes. Thus, *A*_iso_(H*X*) becomes less negative with growing pyramidal distortion (Fig. 2f). Concurrently, *A*_iso_(C1) becomes more positive because the p_*z*_-centered free electron transforms into a more sp^3^ hybridized-type electron, in analogy to ground-state pyramidal 
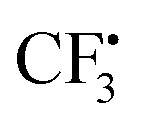
.^47^ Regarding importance matrices of the full hyperfine tensor, the H1C1 bond length has a greater effect on *A*_*x*,*y*_(H1) compared to *A*_*z*_(H1). All principal components are affected by the change in spin population at C and H atoms, but through-space contributions affect *A*_*x*,*y*_ more significantly, as reflected above. For C1, *A*_*x*,*y*,*z*_ importance values are not significantly distinguishable (Fig. S3†).

#### 3.4.2 Ethyl radical

**Fig. 4:**
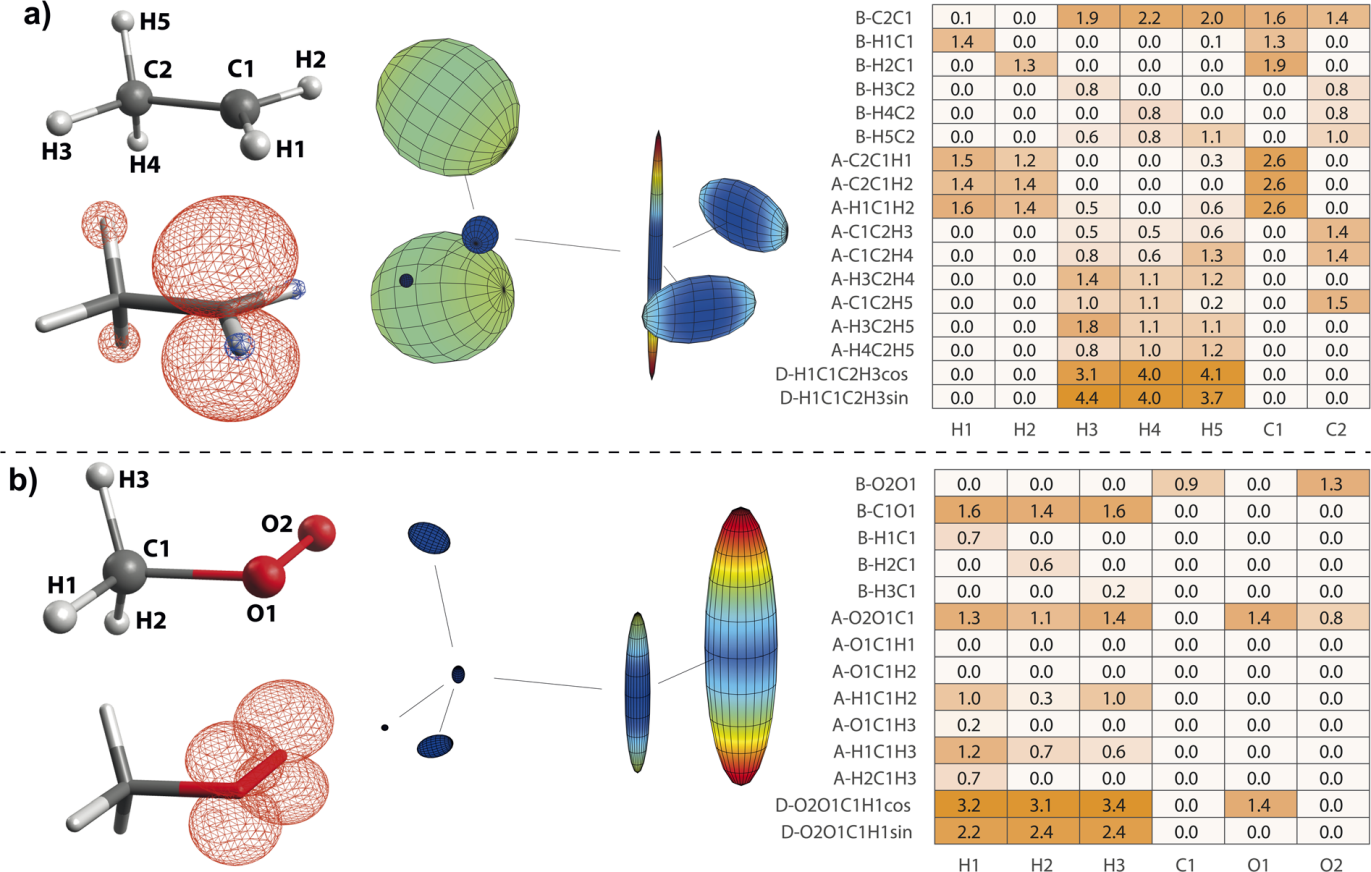
Structure–hyperfine relationships for the ethyl (a) and methyl peroxy (b) radicals. Atomic labels (left top), spin densities (left bottom), and hyperfine coupling tensor visualizations (center) are plotted in analogy to Fig. 2. Importance matrices (right) are given for *A*_iso_ values correlated with bonds (B), angles (A), and dihedrals (D). For dihedrals, importance values for cosine (cos) and sine (sin) values are given.

The ethyl radical 
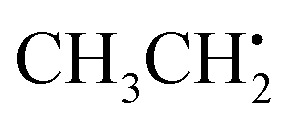
 can be analyzed based on 
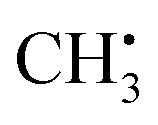
 with one H atom exchanged for a CH_3_ group. The CH_2_ fragment behaves analogous to 
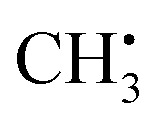
, exhibiting maximum spin population in the C1 p_*z*_ orbital and negative spin density around H1 and H2 (Fig. 4a). Accordingly, non-zero importance values of H1, H2, and C1 are equivalent to the 
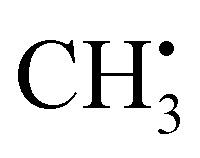
 case, taking into account that all C1-centered angles are now correlated, because C_3_ rotational symmetry is absent and, therefore, each angle matters individually. Exchanging the H residue with CH_3_ stabilizes the ethyl radical *via* hyperconjugation,^13,48^ describing electron delocalization *via* adjacent σ_β-CH_ bonds lying above or below the H1C1H2 plane. This is manifested in large-magnitude ^1^H hyperfine tensors of H4 and H5 and insignificant coupling for H3 lying in the nodal plane in the relaxed structure. However, low-energy CH_3_ rotation renders these atoms (β-H) magnetically equivalent along the MD trajectory. The involved large changes of *A*_iso_ (β-H) depending on the dihedral angle can be identified by a strong correlation in the importance matrix, representing Karplus-like^14^ behavior. Furthermore, non-zero angle importance values define the tetrahedral-to-pyramidal distortion, altering the β-H distance to the p_*z*_ orbital lobes. Interestingly, the C2C1 bond length is also significant for the hyperfine coupling of β-H. This can be attributed to negative spin density around C2 (Mulliken spin population of −0.08), possibly evoked by similar exchange interactions along the C2C1 bond as for H1C1. Further exchange interactions can result in spin polarization of β-H orbitals, as pointed out by *Geoffroy and Lucken*,^49^ challenging the sole attribution of β-H hyperfine values to *McConnell*-type^11,13,44^ hyperconjugation. Accordingly, *A*_iso_(C2) depends strongly on the C2C1 bond but also on the C1C2β-H angles, which affects spin delocalization *via* hyperconjugation toward β-H, thereby offering another path for exchange interactions between β-H and C2.

#### 3.4.3 Methyl peroxy radical

The methyl peroxy radical belongs to the class of alkyl peroxy radicals, which form upon autoxidation of organic compounds. 
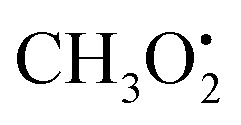
 in particular is relevant for gas-phase atmospheric chemistry as an intermediate in the oxidative decomposition of methane.^50^ Maximum spin density is located in oxygen π-bonded p_*z*_ orbitals with Mulliken spin population of +0.69 and +0.29 for O2 and O1, respectively (Fig. 4b). The ^17^O hyperfine tensors exhibit consistent anisotropy and magnitude. Similar to 
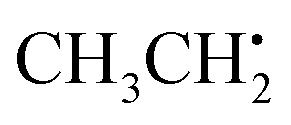
, the π network is further stabilized by σ_CH_ hyperconjugation and *Geoffroy/Lucken*^49^ exchange (*vide supra*), confirmed by large ^1^H importance values regarding the dihedral angle and the C1O1 bond, respectively. In contrast to 
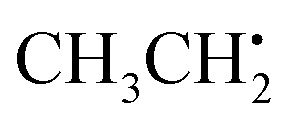
, ^1^H hyperfine coupling changes will not follow approximate C_2_ symmetry with the dihedral, reflecting p_*z*_ symmetry, but are more complex due to increasing (dipolar) interactions when the dihedral approaches ecliptic H*X* and O2. In this position, the O2O1C1 angle dominates the distance of ^1^H to maximum spin density affecting *A*_iso_(H*X*), as deducible from the importance matrix. Also, both ^17^O coupling tensors strongly correlate with O2O1C1, indicating a significant change of electronic structure, possibly involving facilitated hyperconjugation if O2O1C1 is small and/or changes in the π network. *A*_iso_(C1) is comparably small and dictated by the O2O1 bond. This finding can be explained by a cascading effect; if O2O1 is short, π-bonding becomes more efficient, hence the spin population in the O1 p_*z*_ orbital increases, and thus exchange interactions between the O1 p_*z*_ and the C1O1 σ-bond are larger. Although there is no correlation of *A*_iso_(O1) with O2O1 in the displayed importance matrix, an importance value of 1.6 is extracted for *A*_*z*_(O1) (Fig. S4†), which is consistent with the interpretation.

#### 3.4.4 Semiquinone radical

**Fig. 5:**
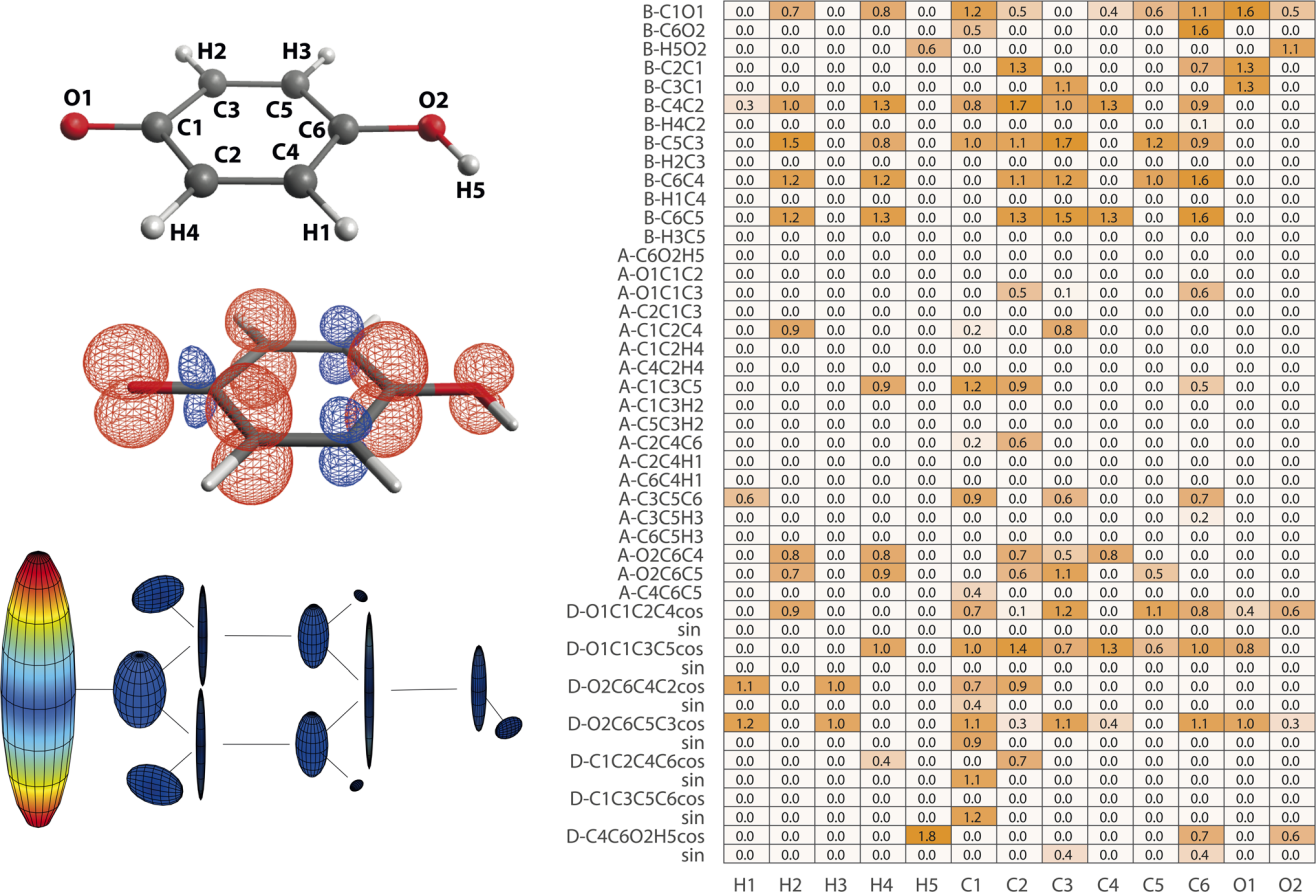
Structure–hyperfine relationships for *p*-benzosemiquinone. (left) Atomic labels, spin densities, and hyperfine coupling tensor visualizations are plotted in analogy to Fig. 2. (right) Importance matrix is given for *A*_iso_ values correlated with bonds (B), angles (A), and dihedrals (D). For dihedrals, importance values for cosine (cos) and sine (sin) values are given.

The family of semiquinones are representative aromatic radicals and serve as electron-transfer agents in biological systems. In comparison to small radicals analyzed thus far, 39 involved structure parameters define the *p*-benzosemiquinone feature space, spanning a 46 × 13 importance matrix (Fig. 5), where machine learning becomes increasingly useful to reduce complexity. Oxygen and carbon atoms form a π network on which spin density is delocalized. The radical is stabilized by the OH group in *para*-position through its positive mesomeric (+M) effect. Mulliken spin population maxima are identified at O1 (+0.38) and at ring carbons in *para*- (+0.32) and *ortho*-position (+0.26) whereas *meta*- (−0.12) and *ipso*-positions (−0.12) are negative. The sign of spin populations is in agreement with simple valence-bond theory.^51^ Ring hydrogens H1–H4 exhibit spin density sign reversal with regard to the directly bonded carbon atoms evoked by exchange interactions.^12,52^

Largest *A*_iso_ is observed for O1 and the importance matrix correlations suggest that its magnitude is predominantly affected by its inclusion into the aromatic ring system; *via* the next-nearest bonds C1O1, C2C1, and C3C1 and *via* its atomic positioning relative to the aromatic plane characterized by the dihedrals O1C1C3C5 and O1C1C2C4. Other deformations within the ring seem to be less impactful, in agreement with findings that the degree of aromaticity remains fairly resistant against medium deformations therein.^53,54^*A*_iso_ values of ring carbons are more complex to define; the multitude of significant structure parameters results in some uncertainty of the algorithm indicated by deviations from C2/C3 and C4/C5 equivalence. Nonetheless, more general statements can be made. In accordance with *A*_iso_(O1), the dihedrals involving O1 position relative to the ring also impact *A*_iso_(C*X*) by alteration of the degree of delocalization into the ring. For that, also the C1O1 bond seems to be significant, especially for *A*_iso_(C1). Within the ring, C–C bond lengths dominate *A*_iso_(C*X*) *via* proposed sensitivity in orbital overlap and exchange, evoking alternating spin density signs around the ring. Inter-ring and ring-hydrogen angles are considered less impacting. Notably, the position of O2 defined by the angle O2C6C4 (and O2C6C5) seems to consistently affect *A*_iso_ of *ortho*- and *meta*-carbons, possibly due to symmetry cancelation along C1C3C5C6 *versus* C1C2C4C6 leading to altered delocalization pathways. A clearer picture arises from ring-hydrogens where symmetry of H1/H3 and H2/H4 is largely intact. It is known that spin density at ring-hydrogens is predominantly caused by *McConnell*^11^ exchange where *A*_iso_ is proportional to the spin density at the adjacent C ring-atom.^12,52^ Therefore, it is no surprise that C–C bond lengths also largely impact *A*_iso_ of ring-hydrogens, which is also evident for H1 and H3 when using a smaller *λ* (ESI, Section E†). Nonetheless, ring-hydrogens are more affected by their position above or below the nodal plane characterized by the correlation with appropriate dihedral angles.

The *para*-positioned OH group is the last fragment to be analyzed. O2 again is highly affected by the C1O1 bond, mediating the degree of delocalization originating from O1. Efficient delocalization is further depending on positioning above or below the aromatic plane, either on the spin density ‘sending’ (O1C1C2C4) or ‘receiving’ side (O2C6C5C3). H5 is positionally more flexible with regard to the aromatic plane (C4C6O2H5), which has a large impact on *A*_iso_(H5). It is dominated by *McConnell*^11^ exchange mediated by the H5O2 bond when within the plane and by participation in the π network above or below the plane.

In addition to the radicals discussed thus far, we also analyzed the tyrosyl radical, structurally similar to semiquinone, and tryptophan-type radical. The corresponding importance matrices are shown in Fig. S5 and S6,† respectively.

### 3.5 Suggestions for application

On a qualitative basis, the introduced importance matrices reveal a multitude of atomic–electronic structure relationships, which typically necessitate elaborate analyses of individual structural changes and their effect(s).^11–13,42–45,47,49,51,52,55^ For small organic radicals, spin density distributions are often conceptually simple to understand and partly transferrable. In contrast, interdependencies in more complicated radicals or paramagnetic species often need to be specifically investigated. For example, systematic computational studies were recently conducted to evaluate the electronic structure of transition metal phosphoroxy compounds,^35,56^ which in part motivated this work. Finding relevant structure relations requires sound chemical intuition and time-consuming variational testing, where the holistic importance matrix approach could speed up the identification of structural relationships and prevent overlooked dependencies. Furthermore, molecular dynamics have often been used to precisely predict *A*_iso_ by averaging a conformational ensemble.^21,22,36,46,57,58^ Apart from mimicking experimental conditions and computing averaged values, MD trajectories could also be concurrently used for the importance matrix approach, given that the needed inputs are already available. The presented procedure can be used for any system, provided hyperfine coupling calculations can be performed with appropriate accuracy.

From a more practical point of view, importance matrices can help answer two types of questions for experimentalists that aid the structure identification of a paramagnetic molecule: (1) *which hyperfine coupling constants do we need to measure to estimate a specific structure parameter?* With pre-experimental awareness of these correlations, EPR experiments can be planned using a design of experiments (DoE) approach. This might involve a decision on whether to investigate liquids or solids, which hyperfine spectroscopy techniques are needed, and which atoms need to be isotope-labeled (*e.g.*^13^C, ^14,15^N, ^17^O). (2) *Which structure parameters could be responsible for a detected change in a specific hyperfine coupling constant?* Changes in structure parameters can have a multitude of reasons and are, therefore, frequently targeted with EPR spectroscopy. In (frozen) solutions, the solvent (or other interacting molecules) is often responsible for structural changes, either implicitly through its dielectric continuum or explicitly, *e.g.*, *via* hydrogen bonding.^59,60^ Temperature was also identified to affect hyperfine couplings,^61^ although again through indirect solvent effects such as viscosity changes. Hyperfine couplings can also be sensitive to structural changes rather remote to the actual radical center (*e.g.*, dihedral-dependent side chain hyperconjugation in semiquinones^57,58^), which might be disregarded in an analysis to reduce complexity/save computational resources or overlooked in the first place.

## 4 Conclusions

We have described an application of the NCA feature selection algorithm to identify relations between atomic configuration and electronic structure in paramagnetic species. The statistical analysis is based on structure parameters and hyperfine tensors of molecular dynamics snapshots, calculated by density functional theory. While conducting MD simulations at experimentally accessible temperatures would be desirable, results suggest that the semi-quantitative information of an importance matrix can be retained at elevated temperatures around 700–1200 K. Adjusting the temperature helps balancing computational costs, and it may also be helpful for sampling a larger configurational space within a particular MD trajectory, as long as the structure of a molecule is not qualitatively altered. Assigning importance quantifiers to structural parameters for *A*_*x*,*y*,*z*,iso_ creates a new perspective to look at, analyze, and understand organic radicals. For the well-characterized methyl and ethyl radicals, importance values reflect known (π)^1^ and (σ_CH_)^2^ exchange interactions and hyperconjugation with β–σ_CH_ bonds. For larger radicals, more subtle correlations are identified, for example that *A*_iso_(C) in the methyl peroxy radical is predominantly dictated by the oxygen–oxygen bond length, or that either exchange or π-delocalization dictates *A*_iso_ of the hydroxy-hydrogen in *p*-benzosemiquinone, depending on the dihedral angle.

The computed importance matrices can help experimentalists in selecting the most sensitive hyperfine couplings for the assessment whether a specific structure parameter is changing. For example, changes can occur *via* H-bonding, which is known to lengthen the C–O bond in *p*-benzosemiquinone.^57^ A sensitive hyperfine coupling constant can also help in identifying confined conformational distributions, as shown for biological tyrosyl and tryptophan radicals.^62,63^ To incorporate all relevant effects, the analysis should be repeated including the explicit molecular environment, *e.g.*, including solvent molecules. We encourage the reader to use and modify the implemented procedure written for ORCA and MATLAB and note that, in principle, also other structure-dependent atomic constants such as quadrupole couplings, chemical shift tensors, *etc.* or isotope variations are suitable for the presented procedure.

## Data availability

The codes used for ORCA calculations are included in the ESI, Section F.† Matlab scripts/functions and trajectory data of the organic radicals investigated herein can be found at https://doi.org/10.26165/JUELICH-DATA/UH0LM2 or at https://github.com/conradsz/StructureHyperfineRelations.

## Author contributions

C. S.: conceptualization, methodology, software, formal analysis, visualization, writing – original draft. R.-A. E.: writing – review & editing, supervision. J. G.: conceptualization, writing – review & editing, supervision.

## Conflicts of interest

The authors declare no conflict of interest.

## Supplementary Material

RA-013-D3RA02476H-s001
