# Supplementary material for: Gauging the importance of structural parameters for hyperfine coupling constants in organic radicals
Source: RSC Adv. 2023 May 12;13(21):14565–74. doi: 10.1039/d3ra02476h (PMC10177955; doi:10.1039/d3ra02476h)
Supplement: RA-013-D3RA02476H-s001 [file RA-013-D3RA02476H-s001.pdf]

Electronic Supplementary Material (ESI) for RSC Advances.
This journal is © The Royal Society of Chemistry 2023

Electronic Supporting Information

to

Gauging the importance of structural parameters for hyperfine coupling constants in organic radicals

Conrad Szczuka [a]*, *Rüdiger-A. Eichel* [a,b], *Josef Granwehr* [a,c]

[a] Institute of Energy and Climate Research (IEK-9), Forschungszentrum Jülich GmbH, 52425 Jülich, Germany
[b] Institute of Physical Chemistry, RWTH Aachen University, 52056 Aachen, Germany
[c] Institute of Technical and Macromolecular Chemistry, RWTH Aachen University, 52056 Aachen, Germany

Table of contents

(A) Parameter choice and optimization ... 2
(B) Selected importance matrices for $A_{x,y,z}$ ... 5
(C) Structure and importance matrix of tyrosyl radical ... 6
(D) Structure and importance matrix of tryptophan-type radical ... 7
(E) Importance matrices with alternative λ values ... 8
(F) Example ORCA input files ... 12

(A) Parameter choice and optimization

Temperature = 300 K, dataset size = 200

	H1, A_{iso}	H2, A_{iso}	H3, A_{iso}	C1, A_{iso}
B-H1C1	1.8	0.0	0.0	1.8
B-H2C1	0.0	1.8	0.0	1.9
B-H3C1	0.0	0.0	1.4	1.4
A-H1C1H2	0.0	0.0	0.9	0.0
A-H1C1H3	0.0	0.0	0.0	0.0
A-H2C1H3	1.0	0.0	0.0	0.0
A-sum	2.2	2.3	2.3	3.2

Temperature = 900 K, dataset size = 200

	H1, A_{iso}	H2, A_{iso}	H3, A_{iso}	C1, A_{iso}
B-H1C1	1.8	0.0	0.0	1.6
B-H2C1	0.0	1.8	0.0	1.7
B-H3C1	0.0	0.0	1.8	1.8
A-H1C1H2	0.0	0.0	1.1	0.0
A-H1C1H3	0.0	1.0	0.0	0.0
A-H2C1H3	1.0	0.0	0.0	0.0
A-sum	2.4	2.4	2.4	3.3

Temperature = 1500 K, dataset size = 200

	H1, A_{iso}	H2, A_{iso}	H3, A_{iso}	C1, A_{iso}
B-H1C1	1.8	0.0	0.0	1.7
B-H2C1	0.0	1.7	0.0	1.6
B-H3C1	0.0	0.0	1.7	1.7
A-H1C1H2	0.0	0.0	1.2	0.3
A-H1C1H3	0.0	1.0	0.0	0.0
A-H2C1H3	1.0	0.0	0.0	0.0
A-sum	3.0	3.1	3.1	4.2

Temperature = 900 K, **dataset size = 25**

	H1, A_{iso}	H2, A_{iso}	H3, A_{iso}	C1, A_{iso}
B-H1C1	1.8	0.0	0.0	0.9
B-H2C1	0.0	1.6	0.0	1.6
B-H3C1	0.3	0.7	1.5	2.5
A-H1C1H2	0.0	0.8	1.2	0.0
A-H1C1H3	0.0	1.2	1.0	0.0
A-H2C1H3	0.0	0.0	0.0	0.0
A-sum	2.2	1.8	2.0	2.8

Temperature = 900 K, **dataset size = 50**

	H1, A_{iso}	H2, A_{iso}	H3, A_{iso}	C1, A_{iso}
B-H1C1	2.1	0.0	0.0	1.6
B-H2C1	0.0	1.7	0.0	1.6
B-H3C1	0.0	0.0	1.4	2.4
A-H1C1H2	0.0	0.3	1.5	0.0
A-H1C1H3	0.0	1.4	0.7	0.0
A-H2C1H3	0.0	0.0	0.1	0.2
A-sum	2.1	1.9	2.1	3.0

Temperature = 900 K, **dataset size = 75**

	H1, A_{iso}	H2, A_{iso}	H3, A_{iso}	C1, A_{iso}
B-H1C1	2.1	0.0	0.0	1.6
B-H2C1	0.0	1.7	0.0	1.6
B-H3C1	0.0	0.0	1.4	2.2
A-H1C1H2	0.0	0.8	1.6	0.0
A-H1C1H3	0.0	1.4	0.7	0.0
A-H2C1H3	0.6	0.0	0.0	0.0
A-sum	2.1	1.9	2.1	2.8

Figure S1. Methyl radical importance matrices. The temperature denotes the molecular dynamics simulation temperature; the dataset size denotes the number of hyperfine calculations, which are performed using snapshots in random steps of 0.5 – 40 fs along the molecular dynamics trajectory. (upper panel) Qualitatively, the importance matrices closely resemble each other. With constant dataset size, especially angle-dependent correlations are more consistent with the symmetry of the molecule at higher temperatures. Comparison of the matrices shows that the ratio of importance values (here, particularly those between bond-related and angle-related) might be changing slightly. (lower panel) Matrices resemble the minimum-error dataset as shown in the main text. Remaining correlations that do not appear for a dataset size of 505 exhibit already comparably small feature weights for a dataset size of 25.

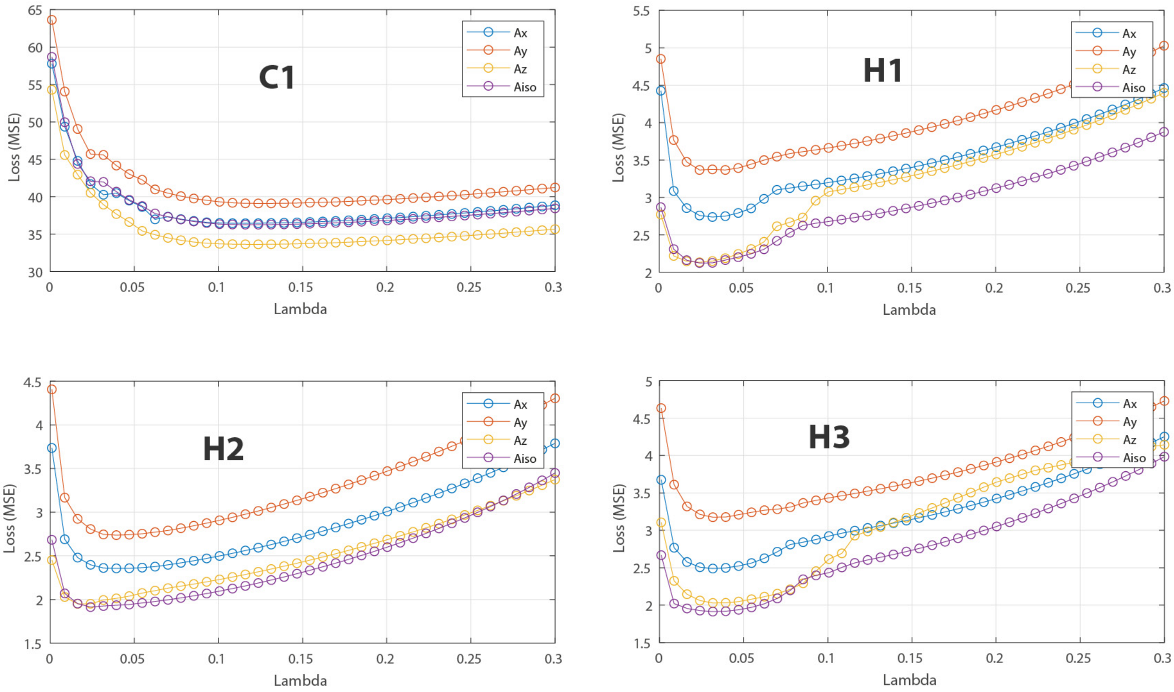

Figure S2. Regularization parameter λ optimization for the methyl radical. λ is plotted against the mean square error (MSE) of the neighborhood components analysis objective function, denoted as loss function in machine learning. The loss function is given by the error estimation between the real outputs and the model. The objective function for minimization is the sum of this loss function and the penalizing regularization term with squared feature weights and the regularization parameter λ. Hyperfine coupling parameter are separately optimized; due to their similarity, their average is computed and given in Supporting Table 1 for each magnetic nucleus.

Table S1. Overview of computational costs for the methyl and semiquinone radicals. The values are extracted from the ORCA output files. Calculation durations reflect the required usage time on the cluster, which includes computational overheads when parallelization is used. The molecular dynamics simulation is the computational bottleneck.

	**Methyl radical**			**semiquinone radical**		
	geometry optimization	molecular dynamics	hyperfine constants	geometry optimization	molecular dynamics	hyperfine constants
calculation duration (hours)	0.01	46	0.006	0.1	384	0.05
number of cores	4	4	4	8	32	4
number of calculations	1	1	505	1	1	1496
total computation time (core days)	0.04	184	12.12	0.8	12288	299.2
computation time (%)	0.020	93.8	6.2	0.006	97.6	2.4

Table S2. Optimized regularization parameters λ for the methyl, ethyl, and methyl peroxy radicals. Magnetically equivalent nuclei are grouped with asterisks. The overall average and mean λ values are used to estimate a reasonable value for all importance matrices. Furthermore, hyperfine coupling constants of the geometry optimized structures are given. For comparison of the computed values, the reader is referred to the following reference: DOI: 10.1080/00268970701604655.

Radical	Nucleus	optimized λ	A_x	A_y	A_z	A_{iso}
methyl	C1	0.12	41.1	41.6	280.3	121.0
	H1*	0.03	-23.9	-62.3	-95.2	-60.5
	H2*	0.03	-20.4	-58.6	-96.3	-58.4
	H3*	0.03	-19.2	-57.1	-97.6	-57.9
ethyl	C1	0.04	2.9	3.8	241.5	82.7
	C2	0.03	-31.9	-32.1	-36.2	-33.4
	H1*	0.02	-28.3	-63.6	-105.1	-65.7
	H2*	0.02	-25.8	-62.0	-102.2	-63.3
	H3**	0.21	-0.7	-1.8	11.5	3.0
	H4**	0.21	115.2	116.8	130.2	120.7
	H5**	0.26	115.3	116.9	130.3	120.8
methyl peroxy	C1	0.01	-11.8	-15.7	-16.4	-14.6
	H1*	0.04	-5.5	-6.3	6.4	-1.8
	H2*	0.05	20.3	21.5	35.1	25.6
	H3*	0.04	26.7	27.7	40.8	31.7
	O1	0.01	23.1	28.9	-158.2	-35.4
	O2	0.01	66.0	68.6	-273.3	-46.3
average		0.07				
median		0.03				

(B) Selected importance matrices for $A_{x,y,z}$

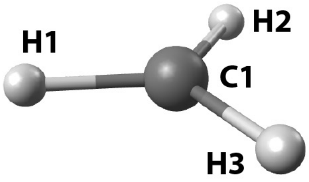

C1

	Ax	Ay	Az	Aiso
B-H1C1	1.8	1.8	1.9	1.8
B-H2C1	2.2	2.2	2.3	2.2
B-H3C1	1.9	1.9	1.9	1.9
A-H1C1H2	0.0	0.0	0.0	0.0
A-H1C1H3	0.0	0.0	0.0	0.0
A-H2C1H3	0.0	0.0	0.0	0.0
A-sum	2.2	2.3	2.1	2.2

Figure S3. Methyl radical importance matrix for all hyperfine tensor principal components of C1 using neighborhood components analysis with $\lambda = 0.05$. The molecular structure is plotted to the left.

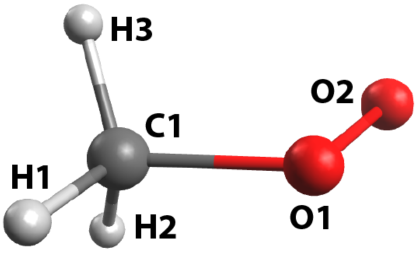

O1

	Ax	Ay	Az	Aiso
B-O2O1	1.2	0.0	1.6	0.0
B-C1O1	0.5	1.5	0.0	0.0
B-H1C1	0.0	0.0	0.0	0.0
B-H2C1	0.0	0.0	0.0	0.0
B-H3C1	0.0	0.0	0.0	0.0
A-O2O1C1	1.2	1.0	1.4	1.4
A-O1C1H1	0.0	0.0	1.1	0.0
A-O1C1H2	0.0	0.0	0.0	0.0
A-H1C1H2	0.0	0.0	0.0	0.0
A-O1C1H3	0.0	0.0	0.9	0.0
A-H1C1H3	0.0	0.0	0.0	0.0
A-H2C1H3	0.0	0.0	0.0	0.0
D-O2O1C1H1cos	0.0	0.0	2.3	1.4
D-O2O1C1H1sin	0.0	0.0	0.5	0.0

Figure S4. Methyl peroxy radical importance matrix for all hyperfine tensor principal components of O1 using neighborhood components analysis with $\lambda = 0.05$. The molecular structure is plotted to the left.

(C) Structure and importance matrix of tyrosyl radical

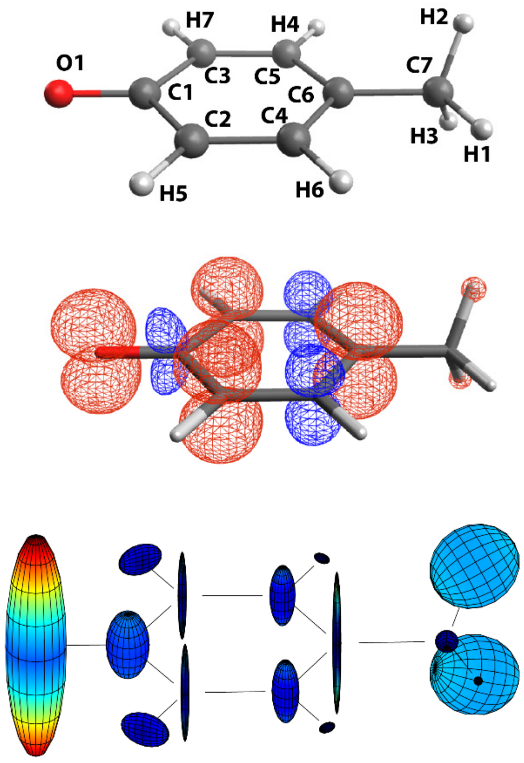

	H1	H2	H3	H4	H5	H6	H7	C1	C2	C3	C4	C5	C6	C7	O1
B-C1O1	0.1	0.8	0.6	0.0	0.0	0.0	0.0	1.3	0.5	0.3	0.9	0.5	1.0	0.9	1.9
B-C2C1	0.9	0.8	0.0	0.0	0.0	0.0	0.0	0.6	1.1	0.5	0.6	0.0	1.1	0.5	1.7
B-C3C1	0.6	0.6	0.3	0.0	0.0	0.0	0.0	0.7	0.4	1.7	0.6	0.0	0.8	0.0	1.4
B-C4C2	0.0	0.1	0.5	0.5	0.7	0.0	1.4	1.0	1.6	0.6	1.2	0.0	0.0	0.0	0.0
B-H7C2	0.0	0.0	0.0	0.0	0.0	0.0	0.0	0.0	0.0	0.0	0.0	0.0	0.0	0.0	0.0
B-C5C3	0.0	0.0	0.0	0.0	1.5	0.5	0.0	0.0	0.0	1.8	0.0	1.6	0.0	0.0	0.0
B-H5C3	0.0	0.0	0.0	0.0	0.0	0.0	0.0	0.0	0.0	0.0	0.0	0.0	0.0	0.0	0.0
B-C6C4	0.1	0.0	0.0	0.0	1.3	0.0	1.5	0.0	1.2	1.4	0.0	1.4	1.6	0.0	0.0
B-H4C4	0.0	0.0	0.0	0.0	0.0	0.0	0.0	0.0	0.0	0.0	0.0	0.0	0.0	0.0	0.0
B-C6C5	0.1	0.6	0.3	0.2	1.4	0.0	1.2	0.0	1.7	1.2	1.3	0.0	1.8	0.0	0.0
B-H6C5	0.0	0.0	0.0	0.0	0.0	0.0	0.0	0.0	0.0	0.0	0.0	0.0	0.0	0.0	0.0
B-C7C6	1.6	1.7	1.6	0.0	0.0	0.0	0.0	0.0	0.0	0.0	0.0	0.0	1.4	1.3	0.0
B-H1C7	0.0	0.0	0.0	0.0	0.0	0.0	0.0	0.0	0.0	0.0	0.0	0.0	0.0	0.0	0.0
B-H2C7	0.1	0.0	0.0	0.0	0.0	0.0	0.0	0.0	0.0	0.0	0.0	0.0	0.0	0.0	0.0
B-H3C7	0.0	0.0	0.0	0.0	0.0	0.0	0.0	0.0	0.0	0.0	0.0	0.0	0.0	0.0	0.0
A-O1C1C2	0.5	0.7	0.6	0.0	0.0	0.0	0.0	0.0	0.0	0.0	0.0	0.0	0.2	0.0	0.0
A-O1C1C3	0.0	1.0	0.8	0.0	0.3	0.0	0.4	0.7	0.8	0.7	0.7	0.9	1.1	0.0	1.1
A-C2C1C3	0.1	0.0	0.1	0.0	0.0	0.0	0.0	1.0	0.0	0.0	0.0	0.0	0.5	0.0	0.0
A-C1C2C4	0.0	0.0	0.0	0.0	1.2	0.0	0.0	0.0	0.0	0.7	0.0	0.0	0.0	0.0	0.0
A-C1C2H7	0.0	0.0	0.0	0.0	0.0	0.0	0.0	0.0	0.0	0.0	0.0	0.0	0.6	0.0	0.0
A-C4C2H7	0.0	0.0	0.0	0.0	0.0	0.0	0.0	0.0	0.0	0.0	0.0	0.0	0.0	0.0	0.0
A-C1C3C5	0.0	0.0	0.1	0.0	0.0	0.0	0.0	0.0	0.9	0.0	0.0	0.0	0.7	0.0	0.0
A-C1C3H5	0.0	0.0	0.0	0.0	0.0	0.0	0.0	0.0	0.0	0.0	0.0	0.0	0.0	0.0	0.0
A-C5C3H5	0.0	0.0	0.0	0.0	0.0	0.0	0.0	0.0	0.0	0.0	0.0	0.0	0.1	0.0	0.0
A-C2C4C6	0.0	0.0	0.0	0.0	0.0	0.0	0.0	0.0	0.6	0.9	0.0	0.0	0.0	0.0	0.0
A-C2C4H4	0.0	0.0	0.5	0.0	0.0	0.0	0.0	0.0	0.0	0.0	0.0	0.0	0.0	0.0	0.0
A-C6C4H4	0.1	0.0	0.0	0.0	0.0	0.0	0.0	0.0	0.0	0.0	0.0	0.0	0.0	0.0	0.0
A-C3C5C6	0.0	0.0	0.0	0.0	0.0	0.0	0.1	0.0	0.6	0.8	0.7	0.0	0.0	0.0	0.0
A-C3C5H6	0.0	0.0	0.1	0.0	0.0	0.0	0.0	0.6	0.2	0.0	0.0	0.0	0.0	0.0	0.0
A-C6C5H6	0.0	0.0	0.0	0.0	0.0	0.0	0.0	0.0	0.0	0.0	0.0	0.0	0.0	0.0	0.0
A-C4C6C5	0.0	0.0	0.7	0.0	0.0	0.0	0.0	0.0	0.2	0.0	0.0	0.0	0.2	0.0	0.0
A-C4C6C7	0.8	0.2	0.2	0.4	0.7	0.0	0.8	0.7	0.9	1.0	0.9	0.6	0.7	0.4	0.5
A-C5C6C7	0.8	1.1	0.9	0.3	0.6	0.3	0.6	0.6	0.7	0.5	0.6	0.8	1.1	0.7	1.8
A-C6C7H1	1.0	0.8	0.8	0.0	0.0	0.0	0.0	0.0	0.0	0.0	0.0	0.0	0.0	0.3	0.0
A-C6C7H2	0.9	0.8	1.2	0.0	0.0	0.0	0.0	0.0	0.0	0.0	0.0	0.0	0.2	0.1	0.0
A-H1C7H2	1.1	0.6	0.9	0.0	0.0	0.0	0.0	0.0	0.0	0.0	0.0	0.0	0.0	0.0	0.0
A-C6C7H3	0.9	0.9	0.8	0.0	0.0	0.0	0.0	0.0	0.0	0.0	0.0	0.0	0.0	0.0	0.0
A-H1C7H3	0.8	1.0	0.9	0.0	0.0	0.0	0.0	0.0	0.0	0.0	0.0	0.0	0.0	0.0	0.0
A-H2C7H3	0.1	0.0	0.3	0.0	0.0	0.0	0.0	0.0	0.0	0.0	0.0	0.0	0.0	0.0	0.0
D-O1C1C2C4cos	0.3	0.6	0.2	0.0	0.0	0.0	0.4	0.7	0.0	0.6	0.9	0.7	0.7	0.6	0.3
sin	1.1	0.9	1.1	0.0	0.0	0.0	0.4	0.6	0.0	0.0	0.2	0.0	1.2	0.0	0.0
D-O1C1C3C5cos	1.0	0.1	0.0	0.6	0.7	0.5	0.5	0.7	1.0	0.6	0.6	0.5	0.6	0.6	0.1
sin	0.0	0.3	0.2	0.0	0.3	0.1	0.0	0.3	0.9	0.5	0.6	0.5	0.2	0.2	0.3
D-C1C2C4C6cos	0.0	0.0	0.0	0.0	0.0	0.0	0.5	0.0	0.7	0.0	0.0	0.0	0.1	0.0	0.0
sin	0.7	0.2	0.7	0.0	0.0	0.0	0.0	0.0	0.0	0.0	0.0	0.0	0.0	0.0	0.0
D-C1C3C5C6cos	0.0	0.0	0.0	0.0	0.0	0.0	0.0	0.0	0.0	0.0	0.0	0.0	0.0	0.0	0.0
sin	0.1	0.0	0.0	0.0	0.0	0.0	0.0	0.0	0.0	0.0	0.0	0.0	0.0	0.0	0.0
D-C2C4C6C5cos	0.2	0.0	0.0	0.6	0.0	0.7	0.0	0.0	0.4	0.0	0.7	0.4	0.4	0.4	0.0
sin	0.7	0.0	0.6	0.0	0.0	0.0	0.0	0.0	0.9	0.0	0.0	0.0	0.1	0.0	0.0
D-C3C5C6C4cos	0.0	0.0	0.0	0.0	0.0	0.0	0.0	0.0	0.0	0.7	0.0	0.0	0.1	0.0	0.0
sin	0.1	0.9	0.0	0.0	0.0	0.0	0.0	0.0	0.0	0.1	0.0	0.0	1.5	0.0	0.0
D-C4C6C7H1cos	2.4	3.2	3.1	0.0	0.0	0.0	0.0	0.0	0.7	0.0	0.0	0.0	1.0	0.0	0.0
sin	3.6	3.2	3.2	0.0	0.0	0.0	0.0	0.0	0.0	0.0	0.0	0.0	0.2	0.0	0.0

Figure S5. Structure–hyperfine relationships for the tyrosyl radical. (left) Atomic labels, spin densities, and hyperfine coupling tensor visualizations are plotted in analogy to Figure 2. (right) Importance matrix is given for A_{iso} values correlated with bonds (B), angles (A), and dihedrals (D). For dihedrals, importance values for cosine (cos) and sine (sin) values are given.

(D) Structure and importance matrix of tryptophan-type radical

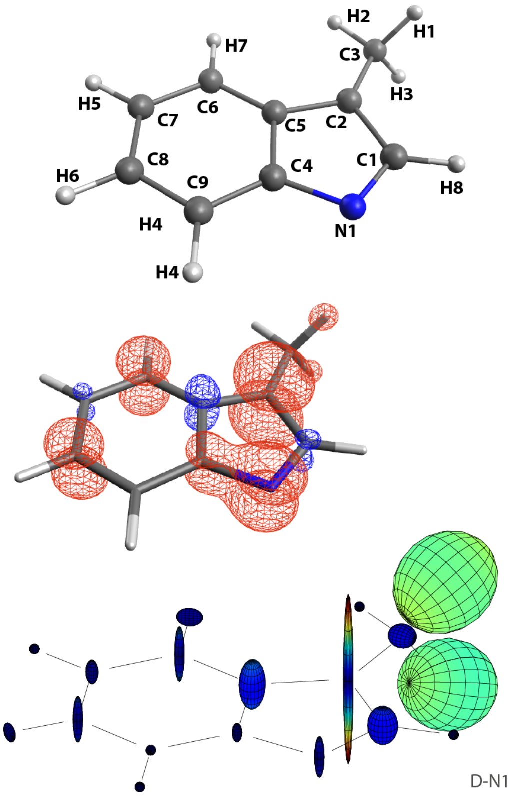

	H1	H2	H3	H4	H5	H6	H7	H8	C1	C2	C3	C4	C5	C6	C7	C8	C9	N1
B-C1N1	0.5	0.1	0.0	1.2	1.1	1.0	0.4	1.5	1.5	0.5	0.4	1.8	1.6	0.5	1.5	1.4	1.4	1.5
B-C4N1	0.0	0.3	0.0	0.4	0.0	0.0	0.0	0.0	0.0	0.0	0.0	0.4	0.0	0.0	0.0	0.0	0.6	0.0
B-C2C1	0.0	0.0	0.0	1.7	1.5	1.0	0.8	1.7	1.8	2.0	0.0	1.6	2.1	1.4	1.6	1.5	1.7	1.6
B-H8C1	0.0	0.0	0.0	0.0	0.0	0.0	0.0	0.0	0.0	0.0	0.0	0.0	0.0	0.0	0.0	0.0	0.0	0.0
B-C3C2	1.6	1.6	1.4	0.0	0.0	0.0	0.0	0.0	0.0	1.5	0.8	0.0	0.0	0.0	0.0	0.0	0.0	0.0
B-C5C2	0.6	0.9	0.0	1.3	1.5	1.5	1.3	1.5	1.7	1.8	0.5	1.6	1.2	1.4	1.7	1.3	1.5	0.9
B-H1C3	0.7	0.0	0.0	0.0	0.0	0.0	0.0	0.0	0.0	0.0	0.0	0.0	0.0	0.0	0.0	0.0	0.0	0.0
B-H2C3	0.0	0.5	0.0	0.0	0.0	0.0	0.0	0.0	0.0	0.0	0.0	0.0	0.0	0.0	0.0	0.0	0.0	0.0
B-H3C3	0.0	0.0	0.5	0.0	0.0	0.0	0.0	0.0	0.0	0.0	0.0	0.0	0.0	0.0	0.0	0.0	0.0	0.0
B-C5C4	0.2	0.0	0.0	0.0	0.0	0.3	0.8	0.0	0.7	0.0	0.0	0.8	0.7	1.0	0.6	0.0	0.0	0.0
B-C9C4	0.0	0.0	0.0	0.0	0.0	0.0	0.4	0.0	0.0	0.0	0.0	0.0	0.0	0.5	0.0	0.0	0.0	0.0
B-C6C5	0.0	0.1	0.0	1.0	0.6	0.9	0.4	0.7	0.7	0.4	0.0	1.1	1.4	1.3	0.8	1.1	1.1	0.6
B-C7C6	0.0	0.0	0.0	1.0	0.0	0.6	0.5	0.9	1.5	0.9	0.0	1.0	0.0	1.6	0.0	1.0	1.2	0.0
B-H7C6	0.0	0.0	0.0	0.0	0.0	0.0	0.0	0.0	0.0	0.0	0.0	0.0	0.0	0.0	0.0	0.0	0.0	0.0
B-C8C7	0.0	0.0	0.0	0.0	0.0	0.6	0.0	0.4	0.9	0.0	0.0	0.8	0.0	0.0	0.3	1.1	0.9	0.0
B-H5C7	0.0	0.0	0.0	0.0	0.0	0.0	0.0	0.0	0.0	0.0	0.0	0.0	0.0	0.0	0.0	0.0	0.0	0.0
B-C9C8	0.0	0.0	0.0	0.0	0.0	0.0	0.5	0.0	0.0	0.0	0.0	0.0	0.0	0.5	0.0	0.9	0.0	0.0
B-H6C8	0.0	0.0	0.0	0.0	0.0	0.0	0.0	0.0	0.0	0.0	0.0	0.0	0.0	0.0	0.0	0.0	0.0	0.0
B-H4C9	0.0	0.0	0.0	0.0	0.0	0.0	0.0	0.0	0.0	0.0	0.0	0.0	0.0	0.0	0.0	0.0	0.0	0.0
A-C1N1C4	0.0	0.0	0.0	0.8	0.6	0.6	0.5	0.0	0.0	0.0	0.0	1.3	0.6	0.8	1.5	1.2	1.1	0.0
A-N1C1C2	0.4	0.0	0.0	0.0	0.0	0.5	0.0	0.0	0.0	0.0	0.0	0.0	0.0	0.0	0.0	0.0	0.0	0.0
A-N1C1H8	0.5	0.0	0.0	0.0	0.0	0.0	0.0	0.0	0.0	0.0	0.0	0.0	0.0	0.0	0.0	0.0	0.0	0.0
A-C2C1H8	0.1	0.0	0.0	0.0	0.0	0.0	0.0	0.0	0.4	0.0	0.0	0.0	0.0	0.0	0.0	0.0	0.0	0.0
A-C1C2C3	0.2	0.9	0.4	0.0	0.0	0.0	0.0	0.0	0.0	0.0	0.0	0.4	0.4	0.0	0.0	0.0	0.0	0.0
A-C1C2C5	0.0	0.0	0.0	0.0	0.0	0.0	0.0	0.0	0.0	0.0	0.0	0.1	0.0	0.0	0.0	0.0	0.0	0.0
A-C3C2C5	0.2	0.2	0.7	0.0	0.0	0.0	0.0	0.0	0.0	0.0	0.0	0.0	0.0	0.0	0.0	0.0	0.0	0.0
A-C2C3H1	0.1	0.2	0.0	0.0	0.0	0.0	0.0	0.0	0.0	0.0	0.0	0.0	0.0	0.0	0.0	0.0	0.0	0.0
A-C2C3H2	0.0	0.5	0.7	0.0	0.0	0.0	0.0	0.0	0.0	0.0	0.4	0.0	0.0	0.0	0.0	0.0	0.0	0.0
A-H1C3H2	1.3	0.9	1.0	0.0	0.0	0.0	0.0	0.0	0.0	0.0	0.6	0.0	0.0	0.0	0.0	0.0	0.0	0.0
A-C2C3H3	0.1	0.7	0.0	0.0	0.0	0.0	0.0	0.0	0.0	0.0	0.3	0.0	0.0	0.0	0.0	0.0	0.0	0.0
A-H1C3H3	1.2	1.0	0.3	0.0	0.0	0.0	0.0	0.0	0.0	0.0	0.0	0.0	0.0	0.0	0.0	0.0	0.0	0.0
A-H2C3H3	0.7	0.1	0.4	0.0	0.0	0.0	0.0	0.0	0.0	0.0	0.5	0.0	0.0	0.0	0.0	0.0	0.0	0.0
A-N1C4C5	0.0	0.0	0.0	0.0	0.0	0.0	0.0	0.8	0.1	0.0	0.0	0.0	0.0	0.0	0.0	0.0	0.0	0.6
A-N1C4C9	0.5	0.4	0.7	0.0	0.0	0.0	0.0	0.0	0.0	0.5	0.0	0.0	0.0	0.0	0.0	0.0	0.0	0.0
A-C5C4C9	0.0	0.2	0.0	0.0	0.0	0.0	0.0	0.0	0.0	0.0	0.0	0.0	0.0	0.0	0.0	0.0	0.0	0.0
A-C2C5C4	0.0	0.0	0.0	0.0	0.0	0.0	0.0	0.0	0.7	0.0	0.0	0.1	0.0	0.0	0.0	0.0	0.0	0.0
A-C2C5C6	0.6	0.0	0.1	0.0	0.0	0.0	0.0	0.0	0.0	0.0	0.0	0.0	0.4	0.0	0.0	0.0	0.0	0.0
A-C4C5C6	0.3	0.2	0.0	0.0	0.0	0.0	0.0	0.0	0.0	0.5	0.0	0.0	0.7	0.0	0.0	0.0	0.0	0.0
A-C5C6C7	0.3	0.0	0.0	0.0	0.0	0.0	0.0	0.0	0.0	0.0	0.0	0.0	0.0	0.0	0.0	0.0	0.0	0.0
A-C5C6H7	0.0	0.2	0.0	0.0	0.0	0.0	0.0	0.0	0.0	0.0	0.0	0.0	0.0	0.0	0.0	0.0	0.0	0.0
A-C7C6H7	0.1	0.1	0.0	0.0	0.0	0.0	0.0	0.0	0.0	0.0	0.0	0.0	0.0	0.0	0.0	0.0	0.0	0.0
A-C6C7C8	0.2	0.0	0.0	0.0	0.0	0.0	0.0	0.0	0.0	0.0	0.0	0.0	0.0	0.0	0.0	0.0	0.0	0.0
A-C6C7H5	0.0	0.0	0.0	0.0	0.0	0.0	0.0	0.0	0.0	0.0	0.0	0.0	0.0	0.0	0.0	0.0	0.0	0.0
A-C8C7H5	0.0	0.0	0.0	0.0	0.0	0.0	0.0	0.0	0.0	0.0	0.0	0.0	0.0	0.0	0.0	0.0	0.0	0.0
A-C7C8C9	0.0	0.0	0.0	0.0	0.0	0.0	0.0	0.0	0.0	0.0	0.0	0.4	0.0	0.0	0.0	0.0	0.0	0.0
A-C7C8H6	0.0	0.0	0.0	0.0	0.0	0.0	0.0	0.0	0.0	0.0	0.0	0.0	0.0	0.0	0.0	0.0	0.0	0.0
A-C9C8H6	0.0	0.0	0.0	0.0	0.0	0.0	0.0	0.0	0.0	0.0	0.0	0.0	0.0	0.0	0.0	0.0	0.0	0.0
A-C4C9C8	0.0	0.1	0.1	0.0	0.0	0.0	0.0	0.0	0.0	0.0	0.0	0.0	0.0	0.0	0.0	0.0	0.0	0.0
A-C4C9H4	0.0	0.0	0.0	0.0	0.0	0.0	0.0	0.0	0.0	0.0	0.0	0.0	0.0	0.0	0.0	0.0	0.0	0.0
A-C8C9H4	0.0	0.0	0.0	0.0	0.0	0.0	0.0	0.0	0.0	0.0	0.0	0.0	0.0	0.0	0.0	0.0	0.0	0.0
D-N1C1C2C3cos	0.0	0.0	0.0	0.0	0.0	0.0	0.0	0.0	0.0	1.2	0.0	0.0	0.8	0.0	0.0	0.0	0.0	0.0
sin	1.8	1.7	1.9	0.0	0.0	0.0	0.0	0.0	0.0	1.2	0.0	0.0	0.0	0.0	0.0	0.0	0.0	0.0
D-N1C4C5C2cos	0.0	0.0	0.0	0.0	0.0	0.0	0.0	0.0	0.0	0.0	0.0	0.0	0.0	0.0	0.0	0.0	0.0	0.0
sin	0.1	0.0	0.5	0.0	0.0	0.0	0.0	0.0	0.0	0.0	0.0	0.0	0.0	0.0	0.0	0.0	0.0	0.0
D-N1C4C9C8cos	0.0	0.8	0.0	0.0	0.0	0.0	0.0	0.0	0.0	0.0	0.0	0.4	0.0	0.0	0.0	0.0	0.0	0.0
sin	0.0	0.1	0.0	0.0	0.0	0.0	0.0	0.0	0.0	0.0	0.0	0.0	0.0	0.0	0.0	0.0	0.0	0.0
D-C1N1C4C5cos	0.0	0.0	0.0	0.0	0.0	0.0	0.0	0.0	0.0	0.0	0.0	0.0	0.0	0.0	0.0	0.0	0.0	0.0
sin	0.0	0.0	0.1	0.0	0.0	0.0	0.0	0.0	0.0	0.0	0.0	0.0	0.0	0.0	0.0	0.0	0.0	0.0
D-C1C2C3H1cos	2.5	3.5	3.5	0.0	0.0	0.0	0.0	0.0	0.0	0.0	0.0	0.0	0.0	0.0	0.0	0.0	0.0	0.0
sin	4.0	3.5	3.6	0.0	0.0	0.0	0.0	0.0	0.0	0.0	0.0	0.0	0.0	0.0	0.0	0.0	0.0	0.0
D-C1C2C5C4cos	0.0	0.0	0.0	0.0	0.0	0.0	0.0	0.0	0.0	0.0	0.0	0.0	0.0	0.0	0.0	0.0	0.0	0.0
sin	0.9	0.9	1.1	0.0	0.0	0.0	0.0	0.0	0.0	1.1	0.0	0.0	0.0	0.0	0.0	0.0	0.0	0.0
D-C2C1N1C4cos	0.0	0.3	0.0	0.0	0.0	0.0	0.0	0.0	0.0	0.9	0.0	0.0	0.0	0.0	0.0	0.0	0.0	0.0
sin	0.0	0.0	0.1	0.0	0.0	0.0	0.0	0.0	0.0	0.0	0.0	0.0	0.0	0.0	0.0	0.0	0.0	0.0
D-C2C5C6C7cos	0.5	0.0	0.0	0.0	0.0	0.0	0.0	0.0	0.0	0.0	0.0	0.2	0.0	0.0	0.0	0.0	0.0	0.0
sin	0.2	0.0	0.0	0.0	0.0	0.0	0.0	0.0	0.0	0.0	0.0	0.0	0.0	0.0	0.0	0.0	0.0	0.0
D-C4C9C8C7cos	0.0	0.0	0.0	0.0	0.0	0.0	0.0	0.0	0.0	0.0	0.0	0.0	0.0	0.0	0.0	0.0	0.0	0.0
sin	0.0	0.0	0.0	0.0	0.0	0.0	0.0	0.0	0.0	0.0	0.0	0.0	0.0	0.0	0.0	0.6	0.0	0.0
D-C5C6C7C8cos	0.5	0.0	0.0	0.0	0.0	0.0	0.0	0.0	0.0	0.0	0.0	0.0	0.0	0.4	0.0	0.0	0.0	0.0
sin	0.1	0.0	0.0	0.0	0.0	0.0	0.0	0.0	0.0	0.0	0.0	0.0	0.0	0.0	0.0	0.0	0.0	0.0
D-C6C7C8C9cos	0.1	0.0	0.0	0.0	0.0	0.0	0.0	0.0	0.0	0.0	0.0	0.0	0.0	0.0	0.0	0.1	0.0	0.0
sin	0.0	0.0	0.0	0.0	0.0	0.0	0.0	0.0	0.0	0.1	0.0	0.0	0.0	0.0	0.0	0.6	0.0	0.0

Figure S6. Structure-hyperfine relationships for the tryptophan-type radical. (left) Atomic labels, spin densities, and hyperfine coupling tensor visualizations are plotted in analogy to Figure 2. (right) Importance matrix is given for A_{iso} values correlated with bonds (B), angles (A), and dihedrals (D). For dihedrals, importance values for cosine (cos) and sine (sin) values are given.

(E) Importance matrices with alternative λ values

$\lambda = 0.025$

CH3, Aiso

	H1	H2	H3	C1
B-H1C1	2.3	0.0	0.0	2.1
B-H2C1	0.0	2.5	0.7	2.7
B-H3C1	0.8	0.0	2.1	2.3
A-H1C1H2	0.3	0.0	2.3	0.0
A-H1C1H3	0.0	2.1	0.8	0.0
A-H2C1H3	2.3	1.0	0.0	0.1
A-sum	1.9	2.0	2.0	2.6

$\lambda = 0.100$

CH3, Aiso

	H1	H2	H3	C1
B-H1C1	1.6	0.0	0.0	1.6
B-H2C1	0.0	1.7	0.0	1.9
B-H3C1	0.0	0.0	1.5	1.6
A-H1C1H2	0.0	0.0	1.6	0.0
A-H1C1H3	0.0	1.5	0.0	0.0
A-H2C1H3	1.6	0.0	0.0	0.0
A-sum	1.3	1.3	1.3	1.9

Figure S7. Methyl radical importance matrices for A_{iso} using alternative λ values.

$\lambda = 0.025$

CH3CH2, Aiso

	H1	H2	H3	H4	H5	C1	C2
B-C2C1	0.8	0.1	2.1	2.1	2.2	2.4	1.6
B-H1C1	1.3	0.4	0.0	0.0	0.0	2.4	0.4
B-H2C1	0.0	1.5	0.0	0.0	0.1	2.1	0.0
B-H3C2	0.0	0.0	0.7	0.0	0.0	0.0	1.0
B-H4C2	0.0	0.0	0.0	0.9	0.1	1.0	1.1
B-H5C2	0.0	0.0	0.7	0.9	1.1	0.7	1.3
A-C2C1H1	2.4	2.3	0.0	0.0	0.4	0.0	0.0
A-C2C1H2	2.3	2.4	0.0	0.0	0.0	0.0	0.0
A-H1C1H2	2.5	2.4	0.8	0.0	0.8	1.0	0.5
A-C1C2H3	0.0	0.0	0.8	0.3	0.6	0.0	1.6
A-C1C2H4	0.0	0.1	1.0	0.9	1.3	0.0	1.7
A-H3C2H4	0.0	0.0	1.4	1.0	1.6	1.3	0.0
A-C1C2H5	0.0	0.1	1.3	1.4	0.3	0.8	1.5
A-H3C2H5	0.0	0.0	2.0	1.2	1.1	1.7	0.0
A-H4C2H5	0.0	0.0	1.1	0.9	1.2	0.0	0.0
D-H1C1C2H3cos	0.9	0.0	3.5	4.6	4.9	0.0	0.0
D-H1C1C2H3sin	0.0	0.0	5.1	4.8	4.1	0.0	0.0

$\lambda = 0.100$

CH3CH2, Aiso

	H1	H2	H3	H4	H5	C1	C2
B-C2C1	0.0	0.0	1.6	1.8	1.8	1.4	1.2
B-H1C1	0.8	0.0	0.0	0.0	0.0	1.4	0.0
B-H2C1	0.0	1.1	0.0	0.1	0.0	1.3	0.0
B-H3C2	0.0	0.0	0.7	0.1	0.0	0.0	0.0
B-H4C2	0.0	0.0	0.0	0.7	0.0	0.0	0.0
B-H5C2	0.0	0.0	0.5	0.6	1.0	0.3	0.4
A-C2C1H1	0.0	0.0	0.1	0.3	0.4	2.0	0.0
A-C2C1H2	0.0	0.0	0.0	0.0	0.0	2.0	0.0
A-H1C1H2	0.0	0.0	0.4	0.0	0.4	2.0	0.0
A-C1C2H3	0.0	0.0	0.5	0.6	0.5	0.5	1.1
A-C1C2H4	0.0	0.0	0.8	0.6	1.1	0.0	1.0
A-H3C2H4	0.0	0.0	1.2	0.8	1.2	0.1	0.0
A-C1C2H5	0.0	0.0	0.9	1.1	0.3	0.1	1.1
A-H3C2H5	0.0	0.0	1.5	1.0	0.9	0.0	0.0
A-H4C2H5	0.0	0.0	0.9	1.0	1.1	0.0	0.0
D-H1C1C2H3cos	0.0	0.0	2.7	3.4	3.5	0.1	0.0
D-H1C1C2H3sin	0.0	0.0	3.7	3.5	3.3	0.0	0.0

Figure S8. Ethyl radical importance matrices for A_{iso} using alternative λ values.

$\lambda = 0.025$

CH3O2, Aiso

	H1	H2	H3	C1	O1	O2
B-O2O1	0.0	0.0	0.0	1.2	0.5	1.6
B-C1O1	1.7	1.5	1.8	0.3	0.9	0.0
B-H1C1	0.9	0.0	0.0	0.0	0.0	0.0
B-H2C1	0.3	0.8	0.0	0.0	0.0	0.0
B-H3C1	0.0	0.0	0.4	0.0	0.0	0.0
A-O2O1C1	1.4	1.2	1.5	0.0	1.7	1.2
A-O1C1H1	0.0	0.6	0.5	0.7	0.0	0.0
A-O1C1H2	0.0	0.0	0.0	0.0	0.0	0.0
A-H1C1H2	1.1	0.4	1.1	0.0	0.0	0.0
A-O1C1H3	0.4	0.0	0.0	0.7	0.0	0.0
A-H1C1H3	1.3	0.9	0.7	0.0	0.0	0.0
A-H2C1H3	0.8	0.6	0.4	0.0	0.0	0.0
D-O2O1C1H1cos	3.5	3.7	4.0	0.0	2.0	0.0
D-O2O1C1H1sin	3.0	2.7	2.7	0.0	0.0	0.0

$\lambda = 0.100$

CH3O2, Aiso

	H1	H2	H3	C1	O1	O2
B-O2O1	0.0	0.0	0.0	0.0	0.0	0.8
B-C1O1	1.4	1.2	1.4	0.0	0.0	0.0
B-H1C1	0.0	0.0	0.0	0.0	0.0	0.0
B-H2C1	0.0	0.0	0.0	0.0	0.0	0.0
B-H3C1	0.0	0.0	0.0	0.0	0.0	0.0
A-O2O1C1	1.2	0.9	1.2	0.0	1.0	0.0
A-O1C1H1	0.0	0.0	0.0	0.0	0.0	0.0
A-O1C1H2	0.0	0.0	0.0	0.0	0.0	0.0
A-H1C1H2	0.7	0.0	0.6	0.0	0.0	0.0
A-O1C1H3	0.0	0.0	0.0	0.0	0.0	0.0
A-H1C1H3	1.1	0.0	0.0	0.0	0.0	0.0
A-H2C1H3	0.1	0.0	0.0	0.0	0.0	0.0
D-O2O1C1H1cos	3.1	2.7	2.9	0.0	0.0	0.0
D-O2O1C1H1sin	1.3	2.1	2.1	0.0	0.0	0.0

Figure S9. Methyl peroxy radical importance matrices for A_{iso} using alternative λ values.

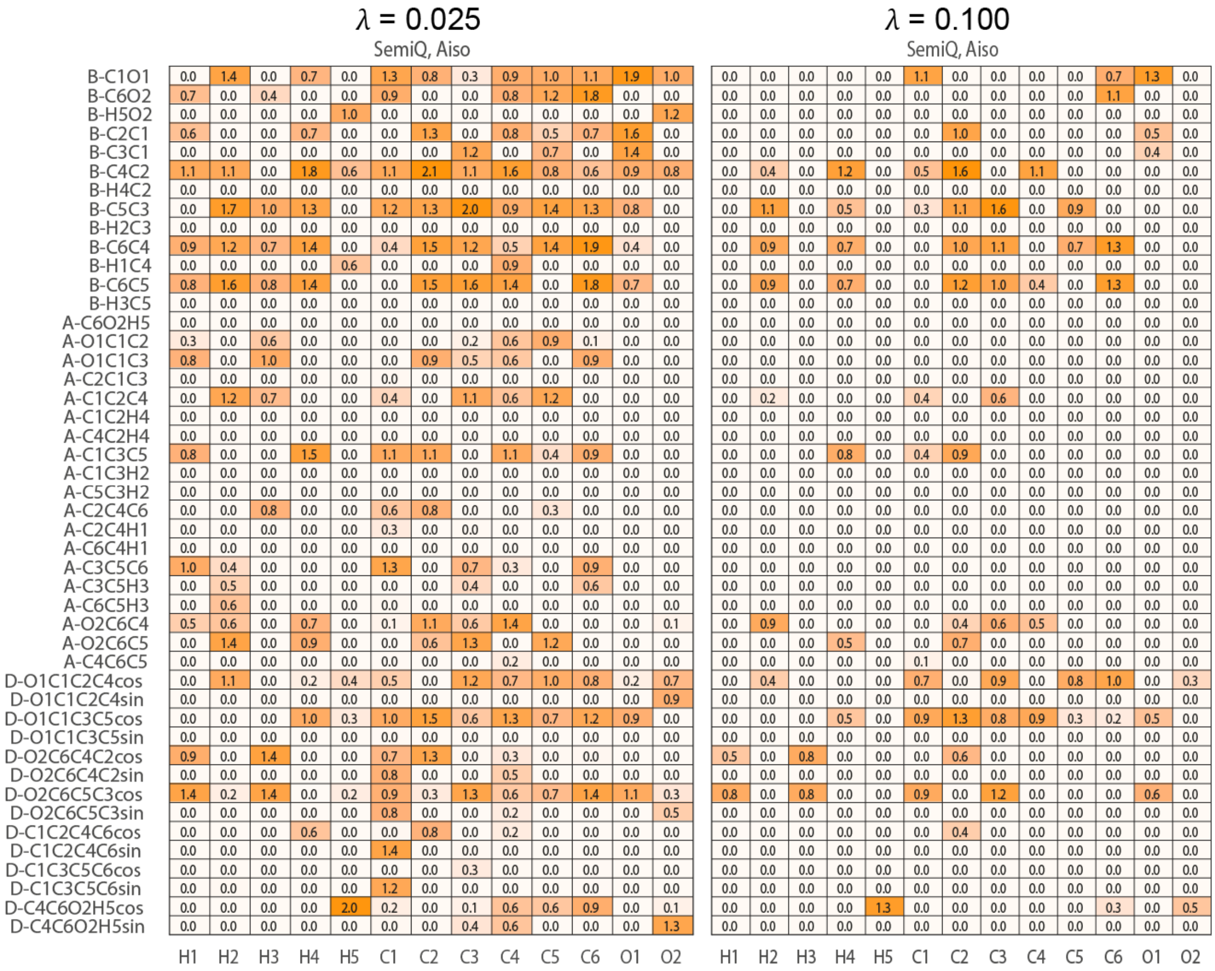

Figure S10. p-benzosemiquinone importance matrices for A_{iso} using alternative λ values.

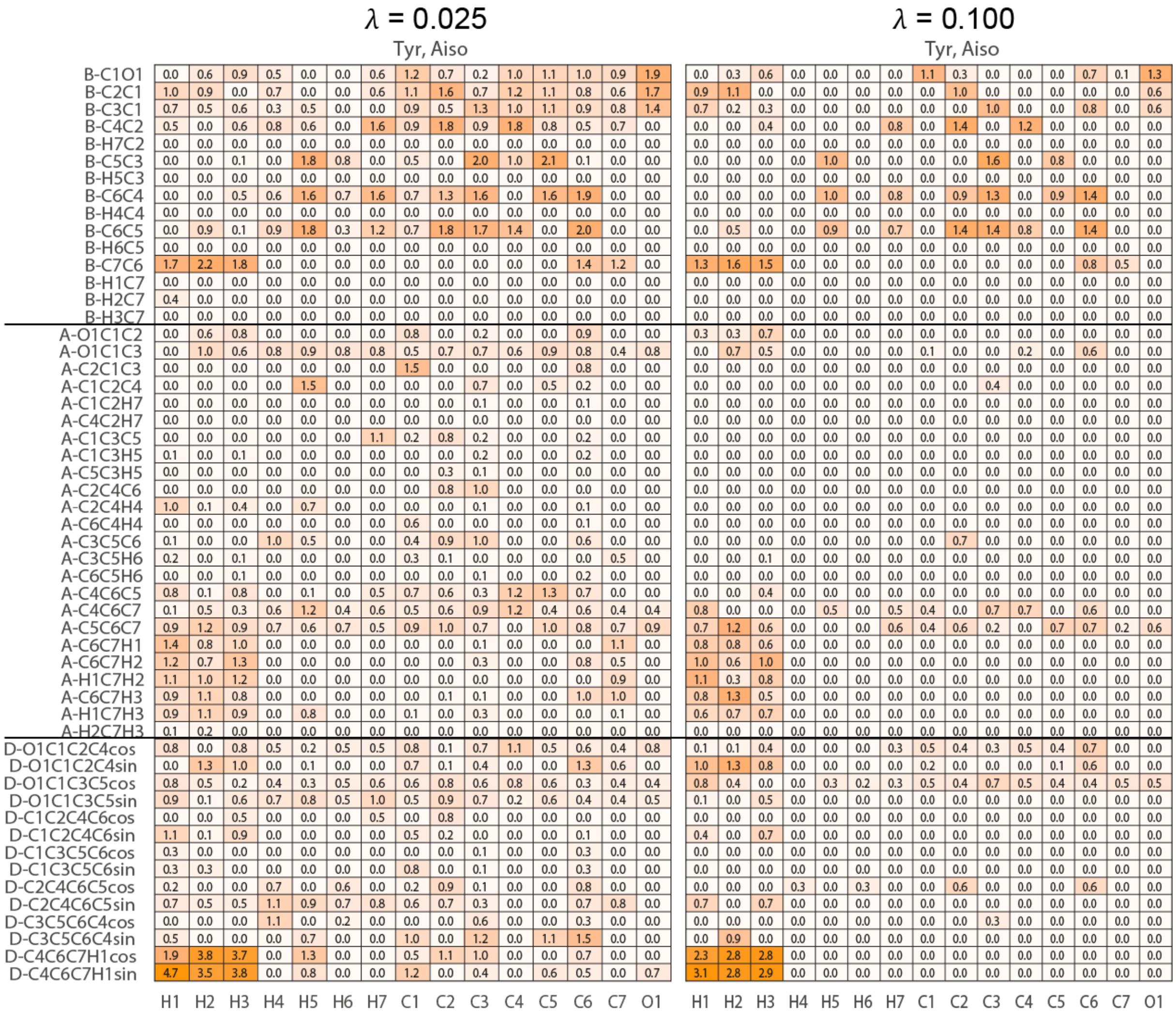

Figure S11. Tyrosyl radical importance matrices for A_{iso} using alternative λ values.

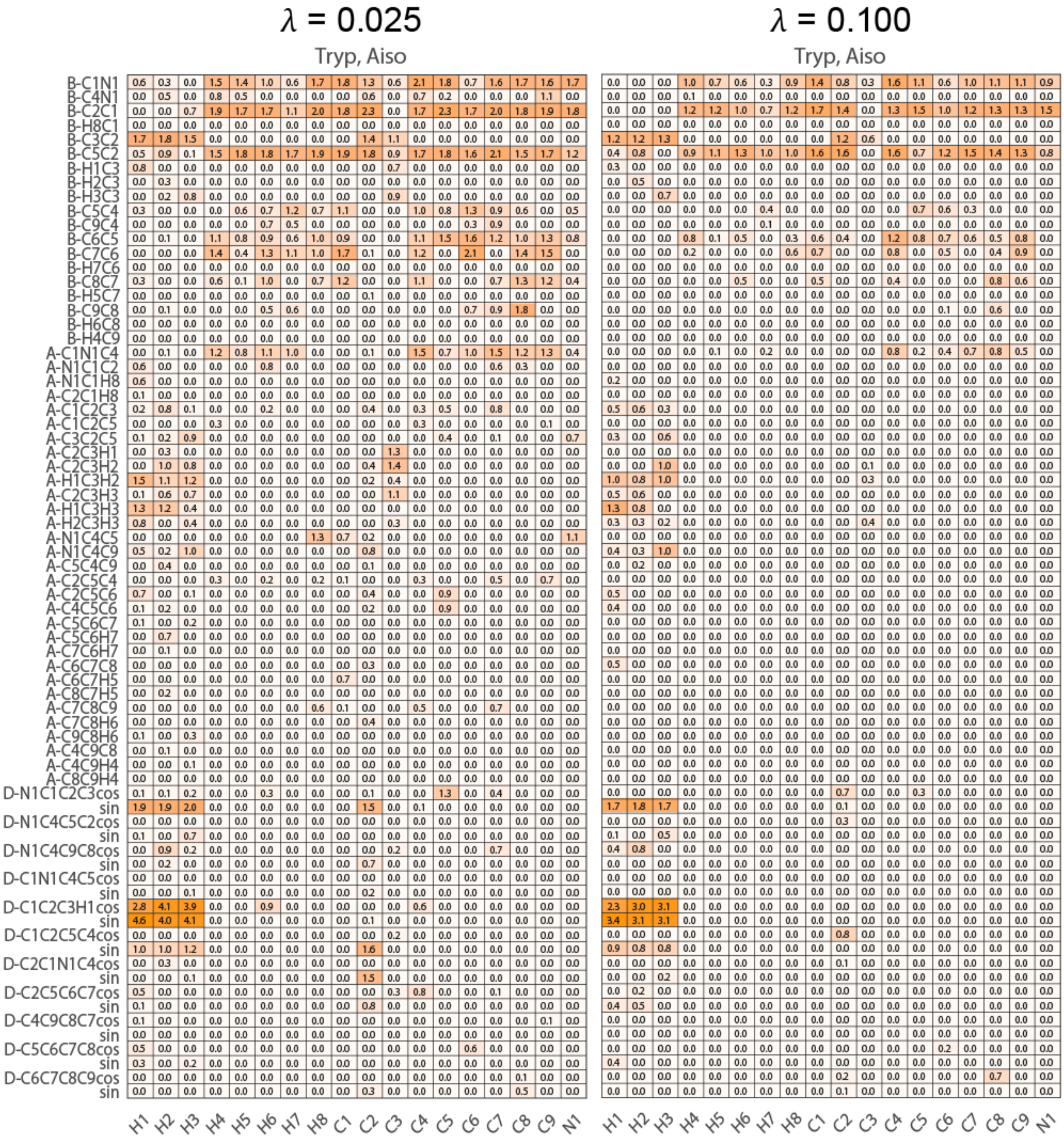

Figure S12. Tryptophan-type radical importance matrices for A_{iso} using alternative λ values.

(F) Example ORCA input files

Code S1. ORCA input file of the methyl peroxy radical for geometry optimization.

```
!B3LYP def2-TZVP OPT
!PAL4

* xyz 0 2
C          4.21793        3.22013       -1.25690
H          4.55418        3.89270       -2.01813
H          4.84511        2.35331       -1.24438
H          4.26345        3.70708       -0.30521
O          3.20026        2.92490       -1.46162
O          2.75217        2.15016       -0.83972
*
```

Code S2. ORCA input file of the methyl peroxy radical for molecular dynamics.

```
! B3LYP def2-TZVP Autoaux
! MD
%pal nprocs 8 end

%md
  randomize                # fully randomize pseudo-random numbers
  initvel 900_K      # initializing velocities
  timestep 0.5_fs          # simulation time step: timestep=sqrt(m)*0.5 fs
  thermostat NHC 900_K timecon 20.0_fs
  dump position stride 1 filename "trajectory.xyz"   # saving
  run 40000                                               # steps
end

* xyz 0 2
  C   4.34381726168830      3.27186822197136     -1.25018445100959
  H   4.65015015611072      3.95799780083515     -2.03769095068005
  H   4.96314893816840      2.37658536558411     -1.24350101227158
  H   4.36290392119334      3.76122436788255     -0.27697283385964
  O   2.98148099158113      2.90340472042924     -1.56806035648166
  O   2.53159873125808      1.97719952329757     -0.74955039569748
*
```

Code S3. ORCA input file of the methyl peroxy radical for hyperfine tensor calculation.

```
!B3LYP EPR-III Autoaux
!PAL4

* xyz 0 2
 C      4.3438172811      3.2718682366     -1.2501844566
 H      4.6501501769      3.9579978186     -2.0376909598
 H      4.9631489604      2.3765853762     -1.2435010178
 H      4.3629039407      3.7612243847     -0.2769728351
 O      2.9814810049      2.9034047334     -1.5680603635
 O      2.5315987426      1.9771995322     -0.7495503991
*
%EPRNMR
        GTENSOR   TRUE
        NUCLEI    = ALL H {AISO, ADIP}
        NUCLEI    = ALL C {AISO, ADIP}
        NUCLEI    = ALL O {AISO, ADIP}
END
```
